# Detection and identification of oil spill species based on polarization information

**DOI:** 10.1371/journal.pone.0291553

**Published:** 2023-11-30

**Authors:** Hongyu Sun, Zhehao Zhao, Qiang Fu, Haodong Shi, Yingchao Li, Di Yang, Jianan Liu, Chao Wang, Huilin Jiang

**Affiliations:** 1 Space Opto-Electronics Technology Institute, Changchun University of Science and Technology, Changchun, China; 2 College of Opto-Electronic Engineering, Changchun University of Science and Technology, Changchun, China; 3 Beijing Institute of Tracking and Telecommunication Technology, Beijing, China; Second Institute of Oceanography Ministry of Natural Resources, CHINA

## Abstract

Aiming at the problem of poor oil identification accuracy in existing oil spill detection technologies, the polarization degree model of oil spill on rough sea surface under different azimuths and zenith angles was established based on Fresnel theory. The analytical expressions of visible light polarization degree in calm and fluctuating water surface were derived respectively, and the polarization degree model of oil spill in reflection space was constructed. The effectiveness of the method and its influence on the polarization distribution of oil spill were analyzed by simulation. A portable turntable was designed to test the polarization characteristics of the experiment, and the visible light polarization detection experiment was carried out. The visible light polarization images of five typical oil spills at different observation azimuth and zenth angles were obtained. The differences in the polarization degrees of different oil species were analyzed, and the correctness of the theoretical model was proved by experiments. The polarization detection experiment of visible light pBRDF was completed, which more intuitively showed the variation law of the polarization characteristics of light reflected by different oil spills in different spatial positions. Using polarization information to distinguish oil species is a useful supplement to the traditional oil spill detection method and has important significance to improve the marine pollution control ability.

## Introduction

Large-scale hydrocarbon pollution caused by marine oil spills poses a serious threat to Marine ecological environment and human production and life [1–10]. At present, the remote sensing monitoring technology of sea oil spill is relatively complete. Different types of sensor systems are mainly established on the air remote sensing platform, satellite remote sensing platform and ship-borne/sea-based/land-based remote sensing platform to achieve all-round three-dimensional monitoring of sea oil slick [11–13].

Polarization detection of marine oil spills has been studied worldwide. Polarization detection is a newly developed detection tool in recent years. Different types of oil spills have different polarization characteristics, so the contrast of oil spills can be improved by polarization detector [11–13]. Under the interference of complex marine environment, polarization information has less influence than the information obtained by traditional intensity detection, which can highlight the characteristics of oil spill and is conducive to the identification and differentiation of targets [14–17]. Shirvany R et al. studied the role of polarization degree through satellite remote sensing images of ships and oil spills under different polarization conditions. But it did not extend the concept of degree of polarization to optics [18]. Sun studied the hyperspectral polarization reflection characteristics of oil slicks on the sea surface, using Daqing crude oil, Jilin crude oil and Dalian Bay seawater as materials to simulate oil film pollution on the sea surface. It is concluded that the inner surface is smaller than the outer surface of the oil film. The study only involves the hyperspectral reflection characteristics of the oil slick on the sea surface, and does not detect the oil slick and distinguish the oil type according to the reflection characteristics [19]. Harmel T and Tonizzo A found that the polarization degree of water surface radiation is related to wavelength, and multi-angle polarization measurement in the visible region is a very effective means [20, 21]. Chenault D B detected oil and diesel oil with a thickness of 50 μm on the water surface with waves, and concluded that the infrared polarization characteristics of oil spill can be used to detect oil spill on the water surface. However, when distinguishing different kinds of light oil or different kinds of heavy oil, the intensity of target polarization radiation is similar, and compared with visible polarization, the contrast of infrared polarization information of the target is lower, so the effect of infrared polarization on oil spill is not obvious [22]. Lai measured the BRDF and pBRDF of three kinds of crude oil and pure water in the visible and near infrared bands indoors by using a scatterometer designed by himself. The results show that it is feasible to use BRDF characteristics for oil detection and oil identification in water background [23]. However, experiments were not conducted in the open area to verify the effectiveness of its conclusions in oil remote sensing. According to the existing literature, there are some limitations in these studies, i.e., how to accurately and effectively detect oil spills and distinguish different types of oil in optical remote sensing. To address this problem, this study further researches and develops methods to combine optical polarization theory and conduct indoor and outdoor polarization measurement tests to analyze the test results, so as to achieve accurate oil spill detection and oil type differentiation.

In order to efficiently distinguish typical types of marine oil spills, based on the polarization detection theory, a rough sea surface oil spill polarization model under different solar zenith angles, observation zenith angles and observation azimuth angles is constructed. In addition, a portable polarization characteristic test turntable is designed to test the polarized bidirectional reflectance distribution function of liquid under external field conditions. The scattering or reflection characteristics of oil spill are tested under simulated real irradiation environment. Finally, the visible light polarization of oil spill is measured at different azimuth and observation angles, which proves the correctness of the theoretical model. The results show that polarization detection can significantly improve the efficiency of offshore oil spill detection.

## Theory and method

Light is basically composed of electromagnetic waves. In the vibration plane of light, the electric vector that plays a major role is generally decomposed into the s component perpendicular to the incident surface of light and the p component parallel to the incident surface of light. When the non-polarized light is reflected at the interface of isotropic smooth media with different refractive indices, the size of the s and p components of the reflected light will change to varying degrees, resulting in polarized light. Fresnel formula can be used to quantitatively calculate the amplitude relationship between reflected light and incident light.


$$r_{s}=\frac{E_{rs}}{E_{is}}=\frac{\sin(\theta_{1}-\theta_{2})}{\sin(\theta_{1}+\theta_{2})}$$ (1)

$$r_{p}=\frac{E_{rp}}{E_{ip}}=\frac{\tan(\theta_{1}-\theta_{2})}{\tan(\theta_{1}+\theta_{2})}$$ (2)

Where *E* is the amplitude; *i* and *r* are incidence and reflection; *θ*_1_ and *θ*_2_ are the incidence angle and the refraction angle.

The light intensity in media with different refractive indices can be expressed by Formula (3):

$$I=\frac{1}{2}\sqrt{\frac{\varepsilon_{0}}{\mu_{0}}}nE^{2}$$ (3)

ε_0_ and μ_0_ are the permittivity and permeability of vacuum, *n* is the refractive index of the medium, and *E* is the amplitude of light.

The reflectance values of the s-light and p-light components in the reflected light are:

$$R_{s}=\frac{I_{rs}}{I_{is}}=\frac{{\left|E_{rs}\right|}^{2}}{{\left|E_{is}\right|}^{2}}=\frac{\sin^{2}(\theta_{1}-\theta_{2})}{\sin^{2}(\theta_{1}+\theta_{2})}$$ (4)

$$R_{p}=\frac{I_{rp}}{I_{ip}}=\frac{{\left|E_{rp}\right|}^{2}}{{\left|E_{ip}\right|}^{2}}=\frac{\tan^{2}(\theta_{1}-\theta_{2})}{\tan^{2}(\theta_{1}+\theta_{2})}$$ (5)

The degree of polarization of the reflected light is:

$$P_{r}=\frac{I_{rs}-I_{rp}}{I_{rs}+I_{rp}}=\frac{\cos^{2}(\theta_{1}-\theta_{2})-\cos^{2}(\theta_{1}+\theta_{2})}{\cos^{2}(\theta_{1}-\theta_{2})+\cos^{2}(\theta_{1}+\theta_{2})}$$ (6)

Combining Formula (6) and Snell ’s law, the degree of polarization of the light reflected by the dielectric surface can be obtained:

$$P_{r}=\frac{\cos^{2}(\theta_{1}-\arcsin(n_{1}\sin\theta_{1}/n_{2}))-\cos^{2}(\theta_{1}+\arcsin(n_{1}\sin\theta_{1}/n_{2}))}{\cos^{2}(\theta_{1}-\arcsin(n_{1}\sin\theta_{1}/n_{2}))+\cos^{2}(\theta_{1}+\arcsin(n_{1}\sin\theta_{1}/n_{2}))}$$ (7)

**Fig 1:**
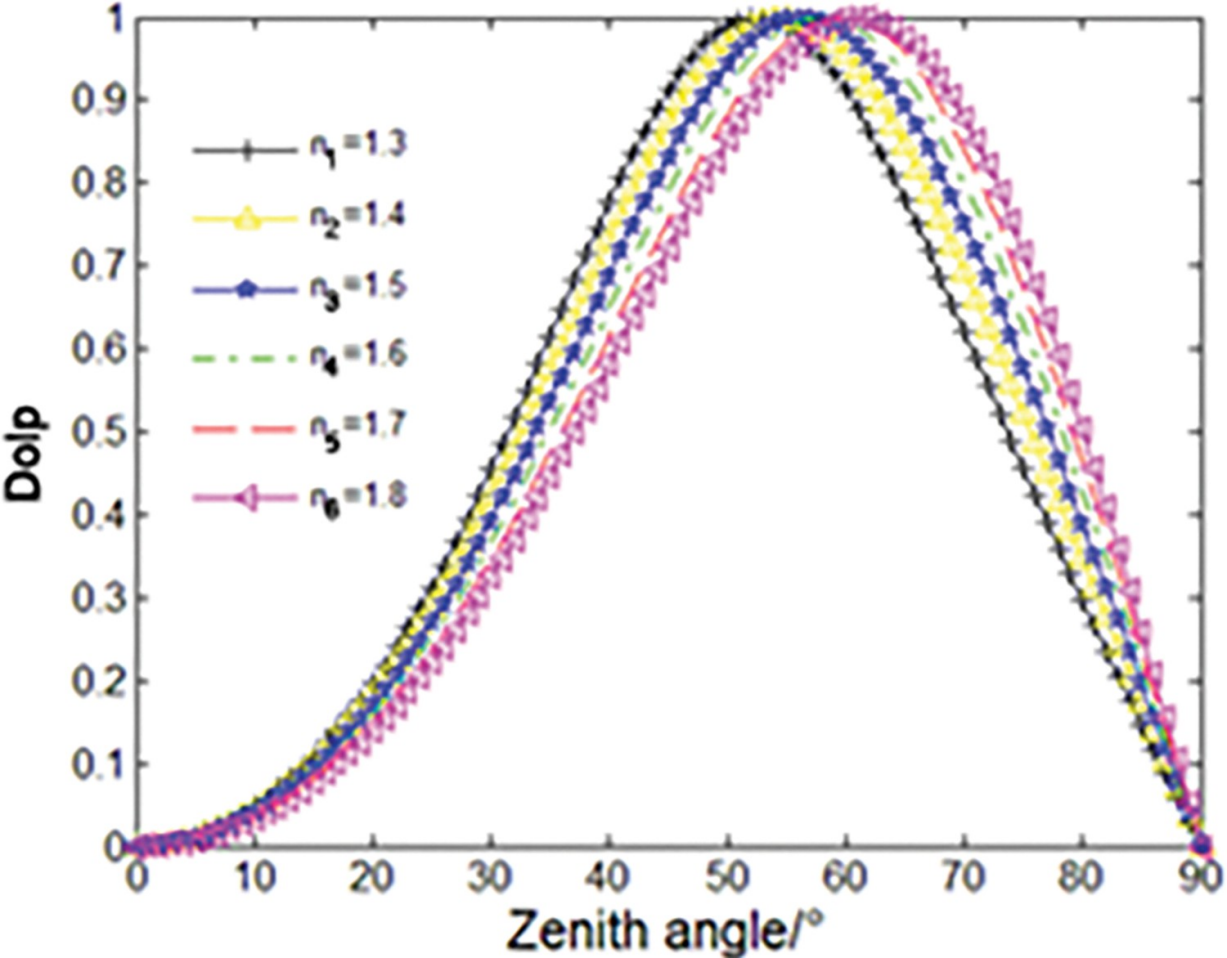
Relationship among incident angle, refractive index and polarization degree of reflected light.

For the gas-liquid interface, we take the air refractive index as 1, the liquid refractive index as 1.3–1.8, the incident angle as 0° to 90°, and use Formula (7) to obtain the relationship between the incident angle of the reflected light, the refractive index and the degree of polarization. Three-dimensional curve fitting is shown in Fig 1. As can be seen from the Fig 1, for each medium, when the refractive index is constant, the degree of polarization of the reflected light increases first and then decreases as the incident angle increases. When the corresponding incident angle is Brewster Angle, the polarization degree of the highest point reflection light is 1. The Brewster angle values of different media are different, generally between 50° and 70°. As the refractive index of the medium increases, the Brewster angle also increases. In addition, the polarization degree is more affected by the angle than the refractive index.

The actual sea surface is not a smooth plane, and spilled oil is polarized as irregular waves are reflected across space. At this time, only relying on the Fresnel formula cannot accurately express the polarization characteristics of the oil spill. Therefore, based on the polarized bidirectional reflectance distribution function (pBRDF) model, this study constructs a polarization model of oil spill reflection on rough sea surface under different solar zenith angles, observation zenith angles and observation azimuth angles [24], as shown in formula (8):

$$F_{r}(\theta_{s},\varphi_{s},\theta_{v},\varphi_{v})=\frac{dL_{pr}(\theta_{s},\varphi_{s},\theta_{v},\varphi_{v})}{dE_{i}(\theta_{s},\varphi_{s})}$$ (8)

Where *θ*_s_ and *φ*_s_ are the solar zenith angle and solar azimuth. *θ*_v_ and *φ*_*v*_ are the observation zenith angle and observation azimuth. *dE*_i_ is incidence irradiance, *dL*_pr_ is reflected irradiance of polarized light. In actual polarization detection, it is difficult to measure irradiance. Typically, the ratio of polarized reflected radiation to incident radiation, the polarized bidirectional reflectance (PBR), is used instead of the PBRDF. PBR can be expressed as Formula (9).


$$R(\theta_{s},\varphi_{s},\theta_{v},\varphi_{v})=\frac{dL_{pr}(\theta_{s},\varphi_{s},\theta_{v},\varphi_{v})}{dL_{i}(\theta_{s},\varphi_{s})}$$ (9)

In order to facilitate the analysis, the fluctuating sea surface is decomposed into several micro-facets with different slopes. The probability distribution of micro-facets with different slopes is affected by the sea surface wind speed, and the formula is expressed as [25]:

$$P(S_{up},S_{cross})=\frac{1}{2\pi \sigma_{up}\sigma_{cross}}\exp(-\frac{1}{2}(\frac{S_{up}^{2}}{\sigma_{up}^{2}}+\frac{S_{cross}^{2}}{\sigma_{cross}^{2}}))$$ (10)

Where *S*_up_ and *S*_cross_ are the slopes of the inclined micro-surface element in the upwind and crosswind directions. *σ*_up_ and *σ*_cross_ are the root mean square of the slope in the upwind direction and the root mean square of the slope in the crosswind direction. $\sigma_{\mathrm{up}}^{2}$ and $\sigma_{\mathrm{cross}}^{2}$ are functions of wind speed, as shown in Eqs (11) and (12) [26]:

$$\sigma_{up,oil}^{2}=0.005+0.00078v$$ (11)

$$\sigma_{cross,oil}^{2}=0.003+0.00084v$$ (12)

**Fig 2:**
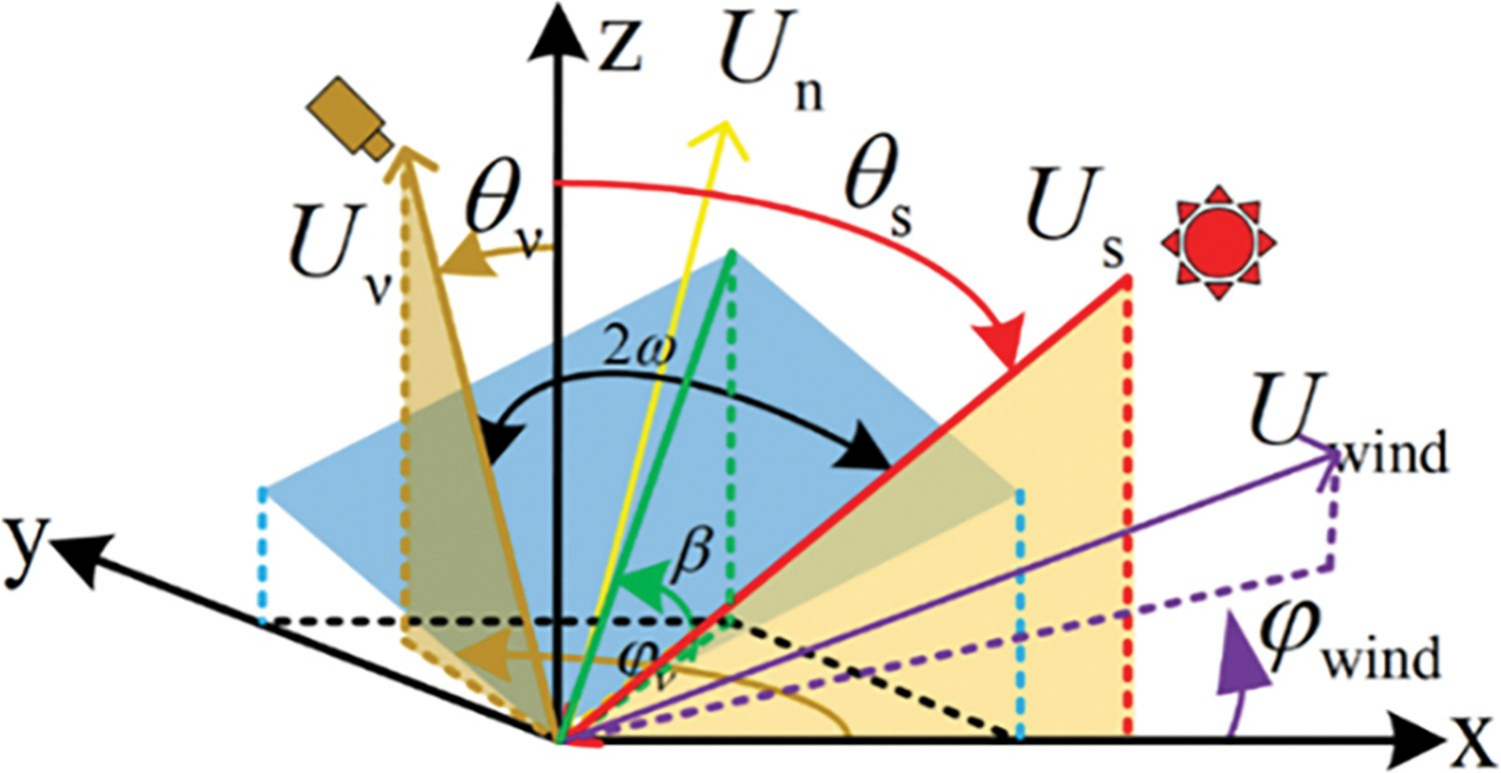
Relationship among parameters of sample reflection space.

In order to analyze the relationship between the elements in the space reflected by the inclined micro-surface element, the coordinate system shown in Fig 2 is established. The incident point on the micro-surface element is taken as the origin, and the *x-axis* direction is defined as the 0° azimuth angle, the *z-axis* direction as the 0° zenith angle, and the *xoz* plane as the incident plane. Therefore, *φ*_s_ = 0°, *φ*_v_ can be regarded as the angle between the observation azimuth and the solar incident azimuth. *U*_*n*_, *U*_*s*_, *U*_*v*_, *U*_*wind*_ are the normal direction of the micro-facet, the direction of light incidence, the direction of the detector observation and the wind direction; *ω* represents the reflection angle of light reflected by the micro-facet mirror, and the angle *β* between the micro-facet and the *xoy* plane; *S*_*x*_ and *S*_*y*_ are the slopes of inclined micro-facet in *x* and *y* directions; the following relationship exists between the parameters:

$$\{\begin{array}{l} S_{x}=\frac{-(\sin\theta_{s}+\sin\theta_{\nu }\cos\varphi_{\nu })}{\cos\theta_{s}+\cos\theta_{\nu }}\\ S_{y}=\frac{\sin\theta_{\nu }sin\varphi_{\nu }}{\cos\theta_{s}+\cos\theta_{\nu }}\\ \tan^{2}\beta =S_{x}^{2}+S_{y}^{2}\\ S_{\mathrm{up}}=S_{x}\cos\varphi_{\mathrm{wind}}+S_{y}\sin\varphi_{\mathrm{wind}}\\ S_{\mathrm{cross}}=S_{y}\cos\varphi_{\mathrm{wind}}-S_{x}\sin\varphi_{\mathrm{wind}}\\ \cos2\omega =\cos\theta_{s}\cos\theta_{\nu }+\sin\theta_{s}\sin\theta_{\nu }\cos\varphi_{\nu } \end{array}$$ (13)

The Formulas (9)–(13) can obtain the reflectivity of p-light and s-light reflected by oil leakage on rough sea surface under different incident angles, observation angles, azimuth angles, wind speeds, wind directions and refractive indexes:

$$R_{\mathrm{p,oil}}(\theta_{s},\theta_{\nu },\varphi_{\nu },\varphi_{\mathrm{wind}},n_{\mathrm{oil}},v)=\frac{r_{\mathrm{p,oil}}^{2}(\omega )P_{\mathrm{oil}}(S_{\mathrm{up}},S_{\mathrm{cross}})}{4\cos^{4}\beta \cos\theta_{\nu }}$$ (14)

$$R_{\mathrm{s,oil}}(\theta_{s},\theta_{\nu },\varphi_{\nu },\varphi_{\mathrm{wind}},n_{\mathrm{oil}},v)=\frac{r_{\mathrm{s,oil}}^{2}(\omega )P_{\mathrm{oil}}(S_{\mathrm{up}},S_{\mathrm{cross}})}{4\cos^{4}\beta \cos\theta_{\nu }}$$ (15)

$$P_{r,oil}=\frac{R_{\mathrm{s,oil}}-R_{\mathrm{p,oil}}}{R_{\mathrm{s,oil}}+R_{\mathrm{p,oil}}}$$ (16)

Combining the Formulas (1), (2), (14)–(16) and Snell ’s law, the degree of polarization of oil spill on rough sea surface at different zenith angles and azimuth angles is obtained:

$$P_{r,oil}=\frac{\cos^{2}(\omega -\arcsin(n_{1}\sin\omega /n_{2}))-\cos^{2}(\omega +\arcsin(n_{1}\sin\omega /n_{2}))}{\cos^{2}(\omega -\arcsin(n_{1}\sin\omega /n_{2}))+\cos^{2}(\omega +\arcsin(n_{1}\sin\omega /n_{2}))}$$ (17)

Where $\omega =1/2\arccos(\cos\theta_{s}\cos\theta_{\nu }+\sin\theta_{s}\sin\theta_{\nu }\cos\phi_{\nu })$. According to the Formulas(14), (15) and (17), although the reflectivity of rough sea surface is affected by incident angle, observation angle, azimuth angle, wind direction, wind speed and medium refractive index, the wind direction and wind speed parameters are reduced when calculating the degree of polarization. Therefore, the degree of polarization is only a function of incident angle, observation angle, azimuth angle and medium refractive index, and has nothing to do with wind direction and wind speed, that is, the degree of polarization is less affected by sea surface fluctuations. It is also theoretically verified that polarization detection has the advantage of being less affected by environmental factors than traditional intensity detection.

**Fig 3:**
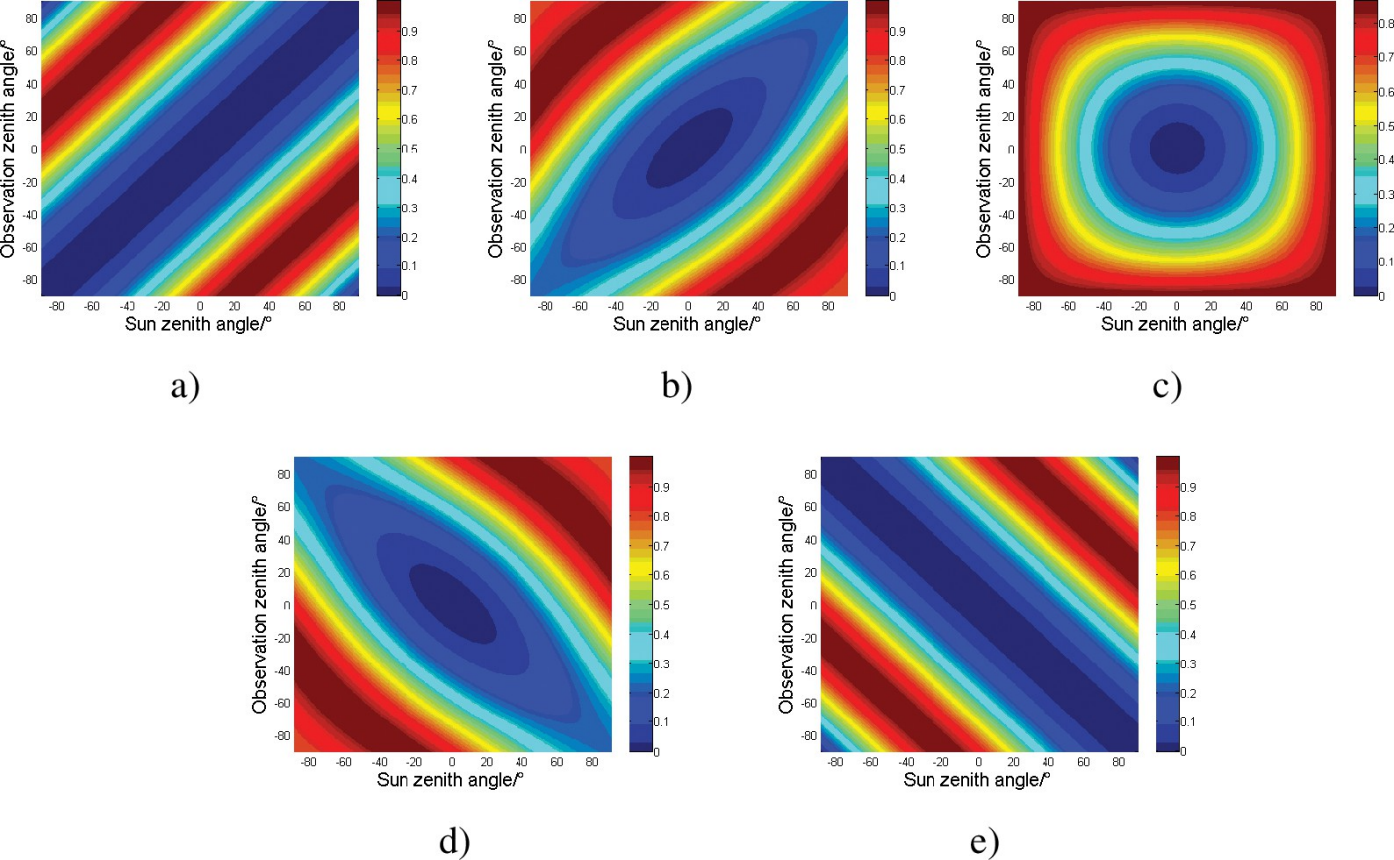
Reflective polarization distribution of oil spill in different spatial positions. a) azimuth 0° b) azimuth 45° c) azimuth 90° d) azimuth 135° e) azimuth 180°.

Next, the relationship between each parameter and the detected polarization degree is analyzed, and the average refractive index of oil spill is 1.42. Fig 3 reflects the reflection polarization distribution of the oil spill at the incident angle -90°~ 90° and observation angle -90°~ 90° when the azimuth angle is taken as 0°, 45°, 90°, 135° and 180°. the color in the Fig 3 corresponding to the degree of color polarization value. It can be seen that due to the symmetry of the spatial position, the polarization images of the two azimuths with the same angle as the 90° azimuth also have a symmetrical relationship. At any azimuth angle, the polarization degree is the lowest when both the incident angle and the observation angle are 0°, and with this point as the center, the near-elliptical direction away from the direction increases first and then decreases. With the increase of azimuth angle, the size of the long axis of the ellipse decreases first and then increases, and the direction will also shift. When the azimuth angle is 90°, the polarization degree distribution is similar to the ring. The degree of polarization in the mirror reflection direction is larger than that in other azimuth angles, and the degree of polarization is higher when the incident angle and the observation angle are equal than when they are not equal. For 0° and 180° directions, the incident angle corresponding to the point with the highest degree of polarization is the target Brewster angle of about 55°.

**Fig 4:**
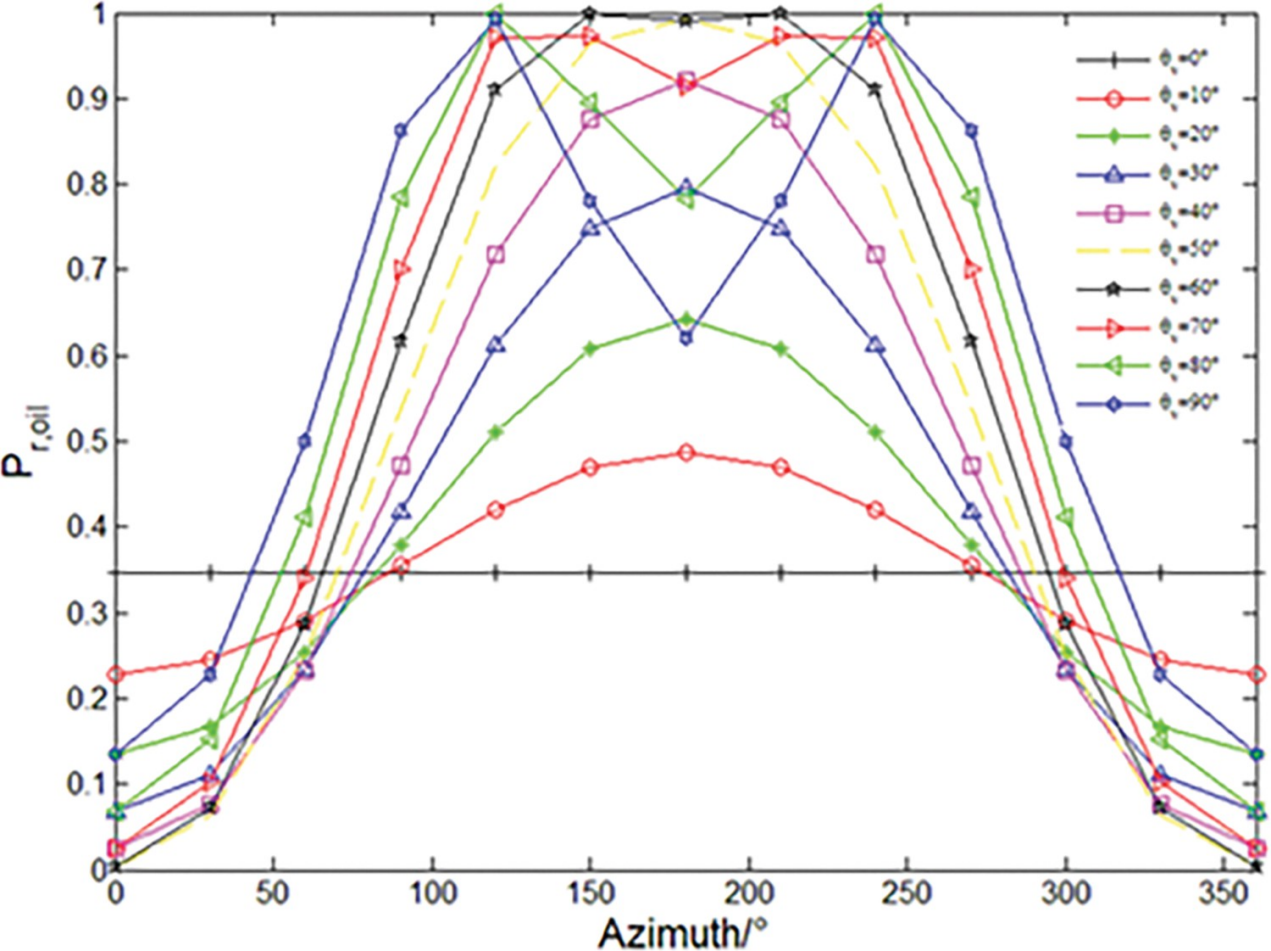
Degrees of polarization for different azimuths and viewing zenith angles at solar zenith angle of 55°.

In order to more intuitively show the influence of observation angle and azimuth angle on the degree of polarization, the incident angle of 55°, azimuth angle of 0° ~ 360° interval of 30° value, observation zenith angle of 0° ~ 90° interval of 10° value is shown in Fig 4 curve. It can be seen from the figure that when the observation angle is less than 55°, the degree of polarization increases with the increase of the observation angle, and increases first and then decreases with the azimuth angle. The overall shape is ’ n ’, and the 180° azimuth reaches the peak. When the observation angle is greater than 80°, the polarization increases and then decreases with the azimuth angle and then increases and then decreases again. The overall shape is ’ M ’, and the peak is symmetrically distributed on both sides of the 180° azimuth angle. Outside the two peaks, the degree of polarization increases with the increase of the observation zenith angle. Inside the two peaks, the degree of polarization decreases with the increase of the observation zenith angle.

### Design of optical detection system for oil spill polarization information

**Fig 5:**
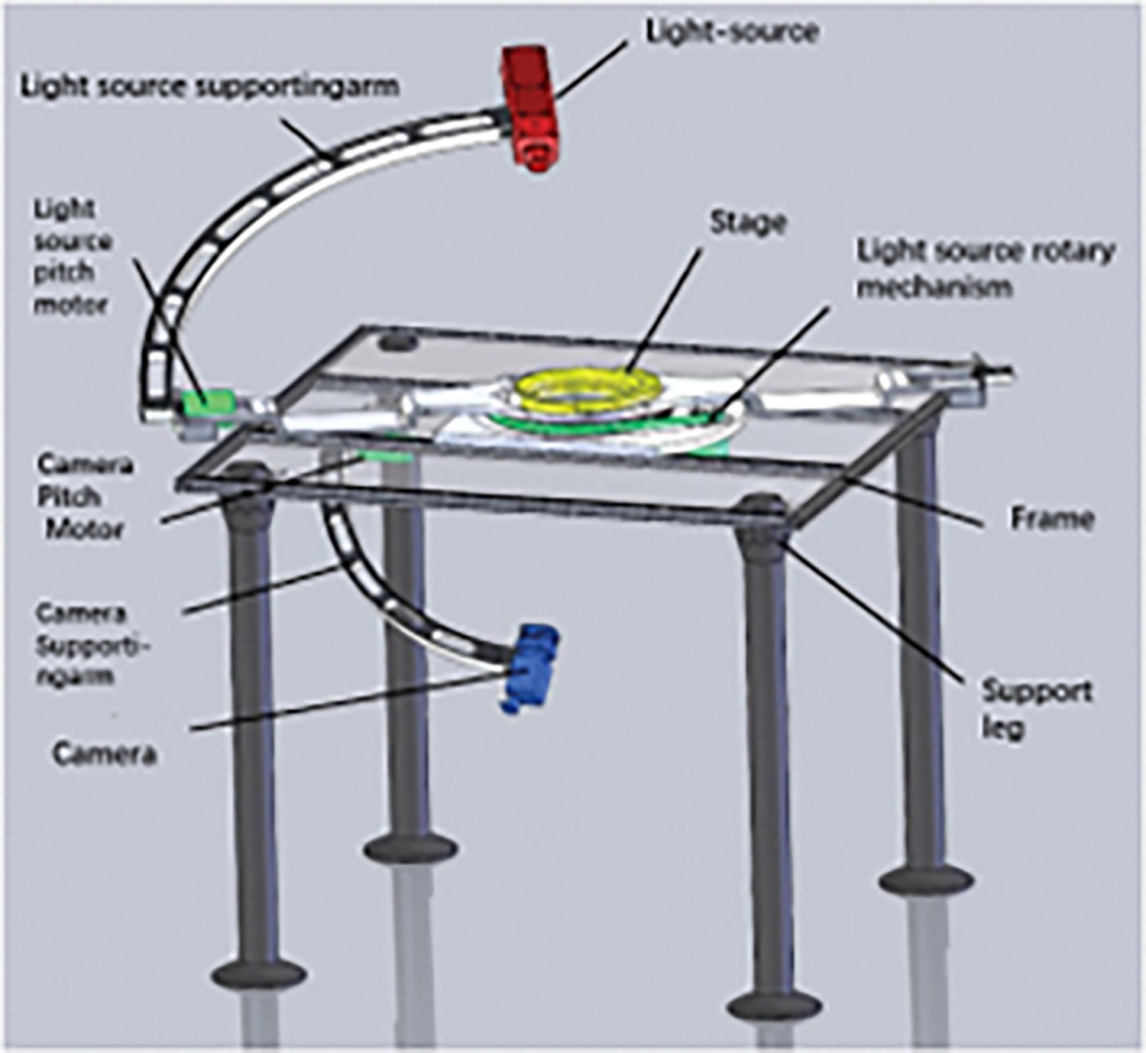
Structure diagram of polarization information optical detection system.

The portable polarization characteristic test turntable is mainly used to test the polarization bidirectional reflection distribution function of the target under the external field condition, and its overall structure is shown in Fig 5. It is mainly composed of darkroom cover, mechanical table, electronic control system, light source and accessories. The guide rail is made of bearing steel, which is used for the movement and positioning of camera and light source, and synchronous tooth belt is installed. Attachments include optical vessels, packaging, installation tooling, etc.; all motion units use RV cycloid reducer motor with high positioning accuracy as power source. Machine can bear more than 150KG, to ensure the stability and reliability of the test platform.

The mechanical table body is mainly composed of semi-circular camera arm, semicircular light source arm, stage, rotary guide rail, camera trolley, light source trolley, rotary trolley, light source, bracket and darkroom cover. The darkroom hood is composed of a folding frame and a light-absorbing curtain, which can effectively reduce the influence of other stray light on the experiment [27–29]; The camera arm is used to support the camera -60°~+60° pitch movement, the light source arm is used to support the light source -60°~+60° pitch movement, and the rotary guide rail is mainly used to support the 360° rotation movement of the camera and light source. The stage is used to carry the experimental sample, which can be adjusted in three dimensions in space, and the hollowing is so that there is a certain space below for transmission measurement; The rotation power of the light source slewing mechanism comes from the high-precision integrated servo motor to ensure its positioning accuracy ≤ 1°; The positioning accuracy of the pitching mechanism of the light source can reach 0.1°; The light source adopts a full-spectrum silicon carbide light source, model Throlab SLS303. The output has an SM1 female thread (Ø1.035"-40) lens sleeve, removable and contains an SM1RR ring for fixing Ø1" or Ø25mm optics up to 18 mm thick, light source spectral range 550 nm—15 μm, output power 4.5 W, collimated beam Ø35 mm, optical drift of 0.01%/0.2% per hour per degree.

**Fig 6:**
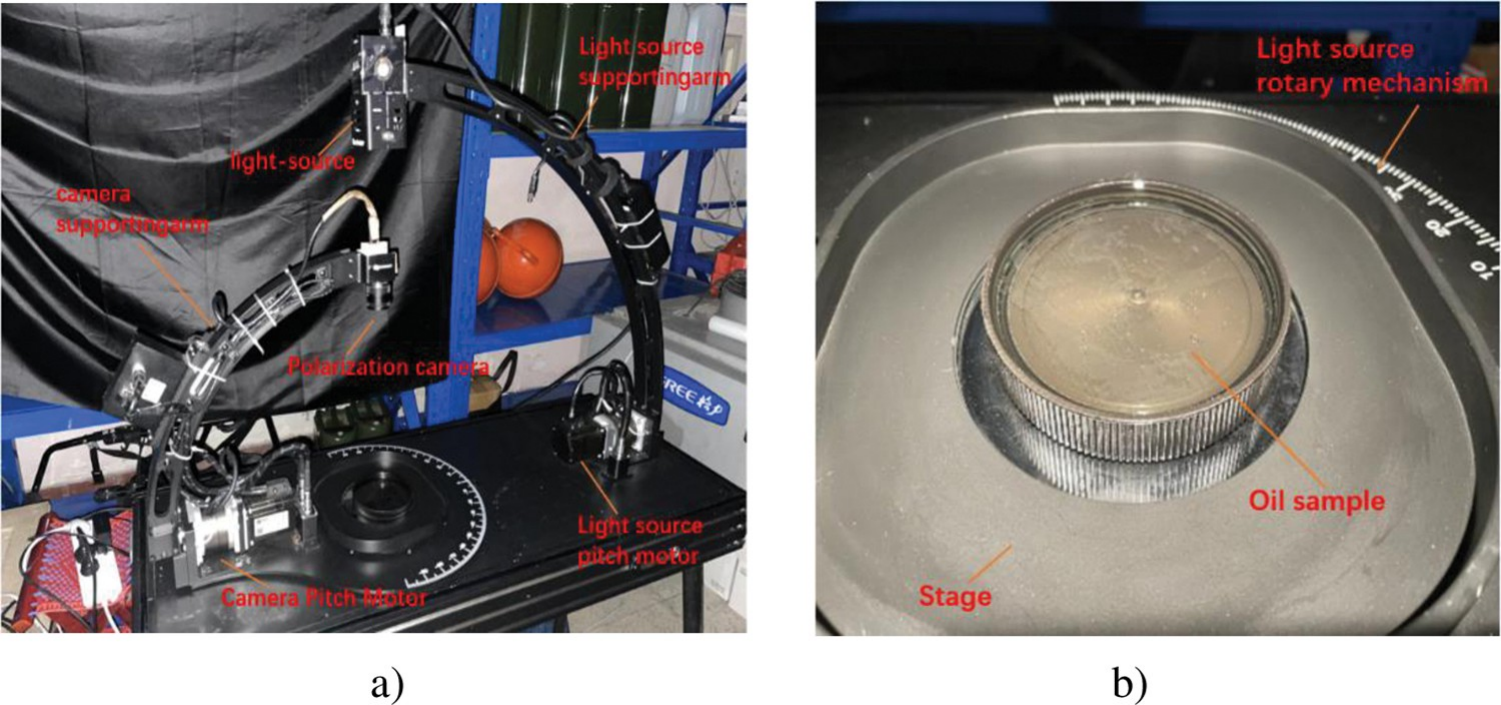
Portable scattering characteristic test turntable.

This structural scheme follows the principles of light structure, convenient transportation, accurate positioning, stable movement and simple structure to carry out the design, the camera is easy to disassemble and assemble, so that the position accuracy of light source rotation, light source pitch and camera pitch can be guaranteed, and the finished product is shown in Fig 6.

**Fig 7:**
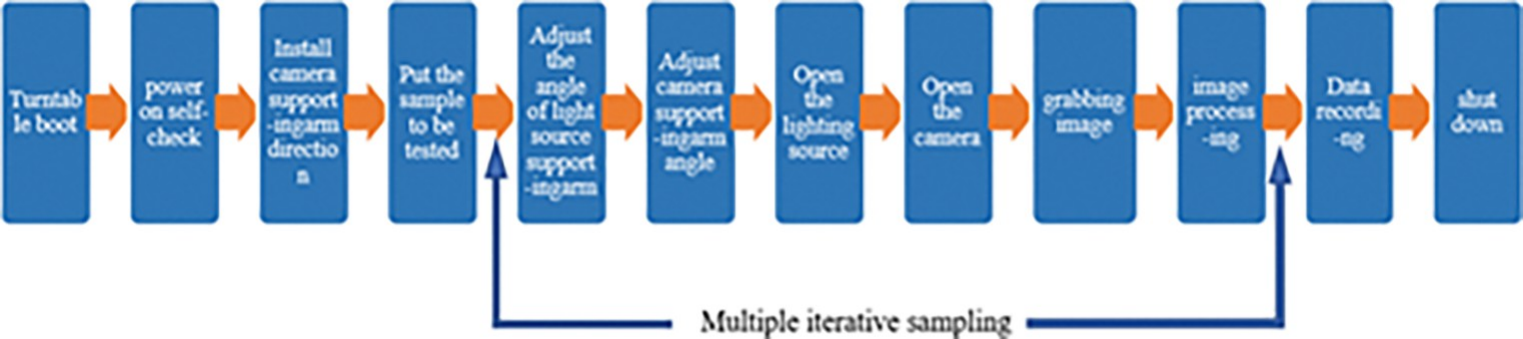
Portable polarization characteristic turntable workflow.

According to the layout of the turntable, the turntable first powers on the work self-test, adjusts the direction of the receiving camera arm according to the sample to be measured, if the sample to be measured is a reflective surface, the camera arm is installed on the upper side of the stage plane, if the sample to be measured is a transparent substance, the camera arm is installed on the lower side of the stage plane; Then, the sample to be measured is placed in the center of the tray, the pitch angle and azimuth angle of the camera arm and the light source arm are adjusted electrically through the control cabinet, and the camera is turned on for image acquisition and the polarization image of the sample to be measured is obtained; Adjust the angle of the light source arm and the camera arm multiple times to sample at different angles; Then image processing, finally data recording and sorting, after the test is turned off and shut down, the workflow is shown in Fig 7.

### Oil spill species discrimination experiment

Experimental scheme and process

Prepare experimental equipment before the test, image acquisition equipment to choose visible polarization camera, and test equipment for calibration to ensure the accuracy of test results; in order to make the data closer to the actual value of the marine environment, the test platform is set up in the outdoor site and the seawater is injected into the water tank to simulate the marine environment, and the acrylic tube is poured into the corresponding oil simulated oil spill area. In order to explore the influence of different azimuth angles and zenith angles on the polarization characteristics of oil spill, the BRDF device is used to realize the camera shooting at different angles in the experiment, and the field of view, focal length and aperture are adjusted to make the target in the center of the field of view and the image clear. Compared with the indoor test, the outdoor environment changes slightly at all times. These changes will make the degree of polarization of the target different, and the size of the difference and the trend of change are difficult to quantify. At this time, if only a set of images will increase the contingency of data, too many shots will lead to poor real-time. Therefore, the number of shots per position is set to five times.

Measure the incident angle of 51° before taking the image, then acquire the visible light 0°, 45°, 90°, 135° polarization direction images of the sample at 0°~360° orientation of 0°~50° zenith angle, and solve the polarization degree and polarization angle images by software. The polarization degree and polarization angle images were calculated by software. After the test is completed, the oil spill in the test is treated to avoid environmental pollution; finally, the acquired image data is processed to eliminate the data that has little effect on the oil type discrimination. The polarization information of the obtained oil spill is extracted and calculated, and the characteristics of different oil types are analyzed to verify the polarization technology. This verifies the feasibility of polarization technology to differentiate oil spill types. Five common marine oil spills, such as fuel oil, palm oil, crude oil, gasoline and diesel oil, were selected as experimental samples, and seawater was selected as a comparative sample. The sample number, sample number and oil parameters are shown in Table 1.

**Table 1:** Oil parameters.

Number	Sample	Density(g/ml)
1	Fuel	0.821
2	Palm oil	0.836
3	Crude oil	0.882
4	Sea water	1.025
5	Gasoline	0.737
6	Diesel	0.835

### Experimental results and analysis

#### Oil classification experiment

**Fig 8:**
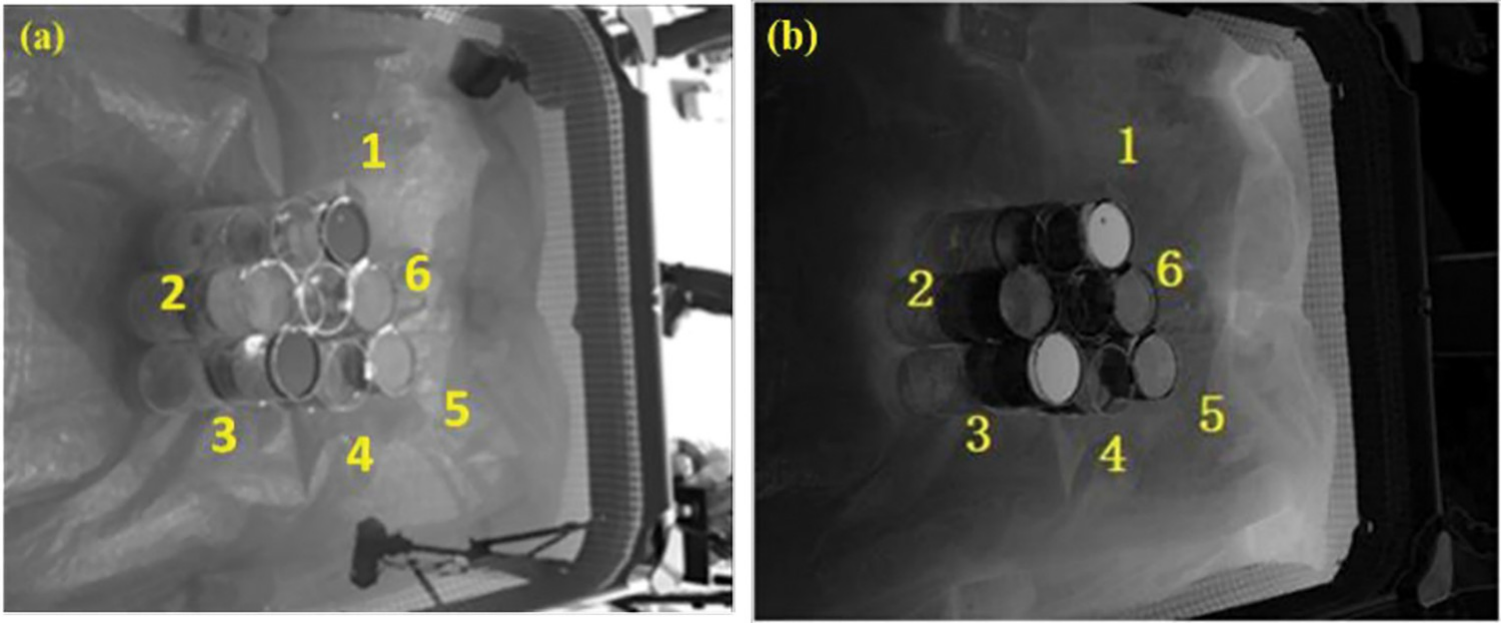
Sideband visible polarization image. a) Visible light intensity image b) Visible light polarization image.

The obtained visible light images were sorted out, and it was found that the polarization angle images had little effect on the differentiation of oil spills, so the rejection was not analyzed. Intensity and polarization data were extracted from visible images of five different sets of samples during side shooting. Fig 8 shows a partially side-shot visible polarization image. Fig 8 corresponds to the serial number of oil species in Table 1.

In the environment, the intensity and polarization degree data of the five sets of the same sample will also have different degrees. Therefore, 95% confidence intervals for the five sets of intensity and polarization data for each sample were calculated to reduce the chance of the data. The 95% confidence interval is calculated as [30]:

$$\left[\mu -1.96\times \frac{\sigma }{\sqrt{n}},\mu +1.96\times \frac{\sigma }{\sqrt{n}}\right]$$ (18)

Where *μ* is the mean, *σ* is the standard deviation, and *n* is the number of groups of experimental data. In order to show the degree of differentiation between the oil species, the contrast of each oil species relative to the sea water is calculated with sea water as the reference and the mean value of the confidence interval as the reference. Define the intensity contrast as:

$$C_{I}=\frac{I_{oil}-I_{seawater}}{I_{seawater}}$$ (19)

The polarization contrast ratio of visible light is:

$$C_{P}=\frac{P_{oil}-P_{seawater}}{P_{seawater}}$$ (20)

Where *C* represents contrast, *I* represents intensity, and *P* represents polarization.

**Fig 9:**
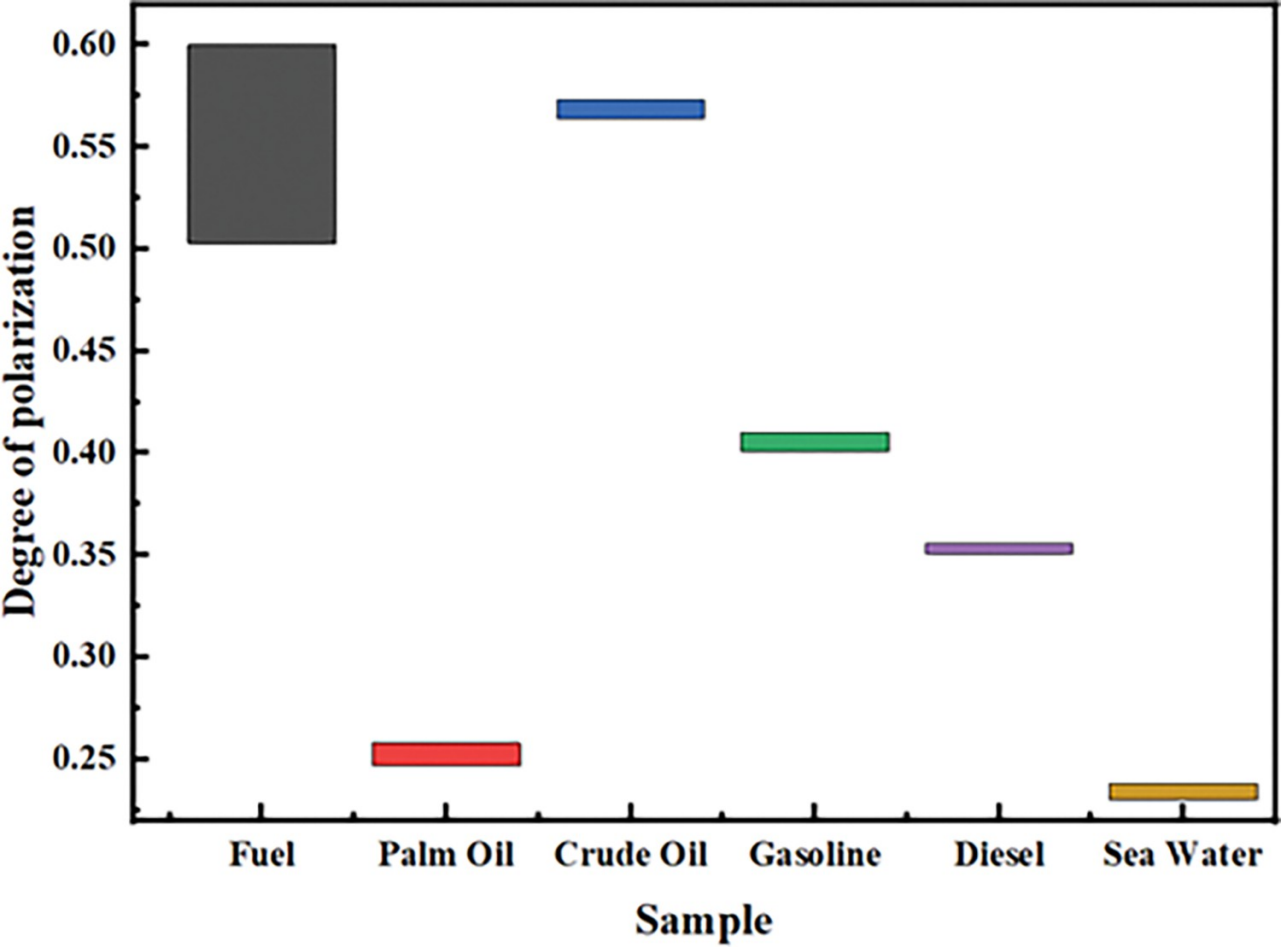
95% confidence interval of each sample in side pat.

Table 2 shows the 95% confidence interval data for visible light intensity and polarization for each sample. It can be seen from Table 2 that the visible light intensity reflected by each sample from high to low is gasoline, diesel, palm oil, seawater, crude oil and fuel oil. The 95% confidence intervals of the intensity of gasoline, diesel and palm oil are [156.71, 158.53], [148.45, 151.37] and [140.86, 143.42], and there is no intersection, and there are significant differences between them. These three oil leakage events can be distinguished by intensity images. The intensities of fuel oil, crude oil and seawater in the 95% confidence interval are [100.83,102.05], [102.11,104.31], [103.05,104.81], respectively. There is little difference or even intersection, so it is difficult to distinguish fuel oil, crude oil and seawater in the intensity image. The degree of polarization of the reflected light from high to low for each sample is fuel oil, crude oil, gasoline, diesel, palm oil and seawater. The 95% confidence interval corresponding to the degree of polarization is [0.593,0.600], [0.564,0.573], [0.401,0.410], [0.351,0.356], [0.247,0.258], [0.230,0.238], and there is no intersection, which can be used to distinguish oil species. When images are taken from a side view, the polarization contrast of each oil relative to seawater is usually higher than the visible intensity contrast. As shown in Fig 9, the polarization information of oil and crude oil, which is difficult to distinguish in intensity image, also shows obvious difference.

**Table 2:** 95% confidence interval of each sample in side pat.

Number	Sample	Strength	Degree of polarization
1	Fuel	[100.83, 102.05]	[0.593, 0.600]
2	Palm oil	[140.86, 143.42]	[0.247, 0.258]
3	Crude oil	[102.11, 104.31]	[0.564, 0.573]
4	Sea water	[103.05, 104.81]	[0.230, 0.238]
5	Diesel	[148.45, 151.37]	[0.351, 0.356]
6	Gasoline	[156.71, 158.53]	[0.401, 0.410]

**Fig 10:**
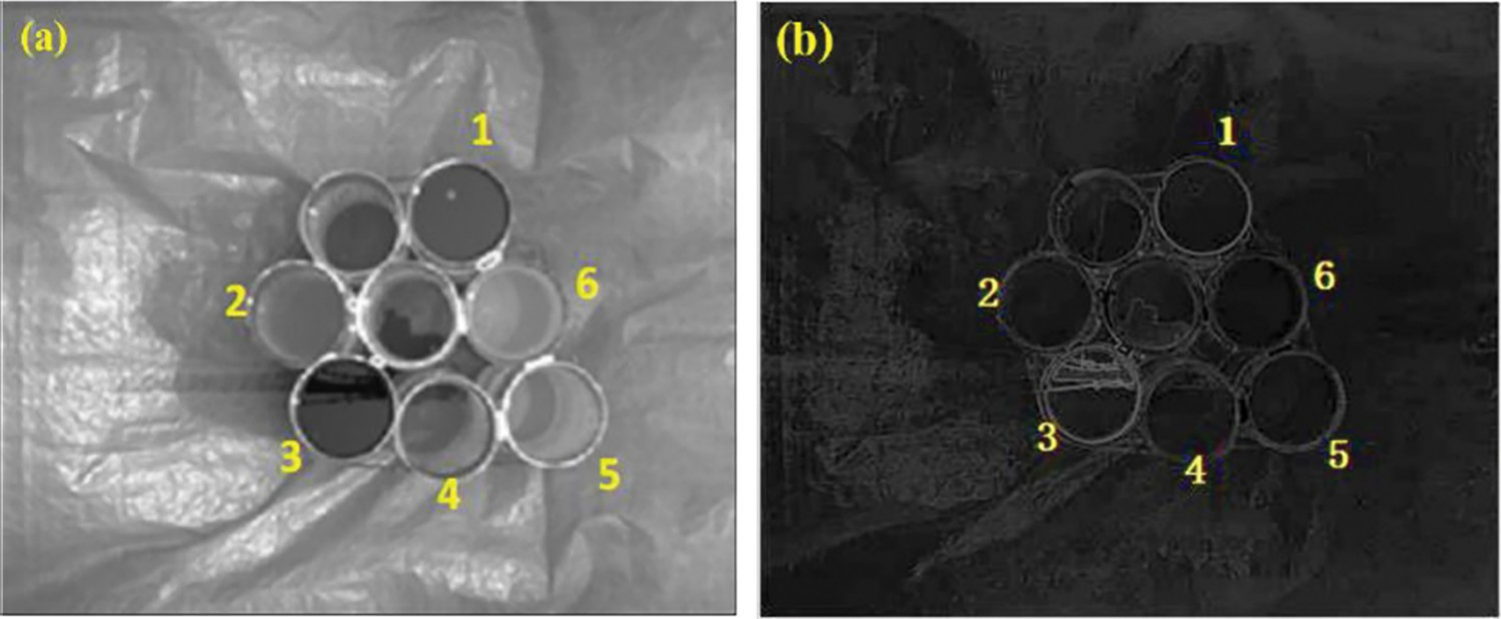
Undershoot visible light image. a) Visible light intensity b) Visible light polarization.

Fig 10 shows the sample image taken from the top view. The 95% confidence interval data for the visible light intensity and polarization of each sample image taken from the top view are shown in Table 3.

**Table 3:** The 95% confidence interval of the intensity and polarization of the visible light of each sample during the top shot.

Number	Sample	Strength	Degree of polarization
1	Fuel	[87.71, 89.03]	[0.0154, 0.0199]
2	Palm oil	[99.60, 102.30]	[0.0173, 0.0209]
3	Crude oil	[54.71, 55.69]	[0.0444, 0.0471]
4	Sea water	[97.70, 100.40]	[0.0168, 0.0212]
5	Diesel	[142.97, 145.09]	[0.0122, 0.0148]
6	Gasoline	[133.18, 134.12]	[0.0157, 0.0197]

As can be seen from Table 3, the visible light intensity reflected by each sample is roughly listed in order of diesel fuel, gasoline, palm oil, seawater, fuel oil and crude oil, from highest to lowest. The 95% confidence intervals for diesel, gasoline and palm oil intensity were [142.97,145.09], [133.18,134.12] and [99.60,102.30], with no intersection and significant differences. The distinction between these three oils is achieved through an intensity image. The 95% confidence intervals for fuel oil, crude oil, and seawater strength are [87.71,89.03], [54.71,55.69], and [97.70,100.40]. It shows that the differences are very small or even crossed. It is difficult to distinguish between fuel oil, crude oil, and seawater in the intensity image.

By comparing side view image and top view image, the polarization degree of visible light is obviously affected by viewing angle. And the degree of polarization taken from the top view is much smaller than that taken from the side view. Oil types are more easily distinguished in the side view.

**Fig 11:**
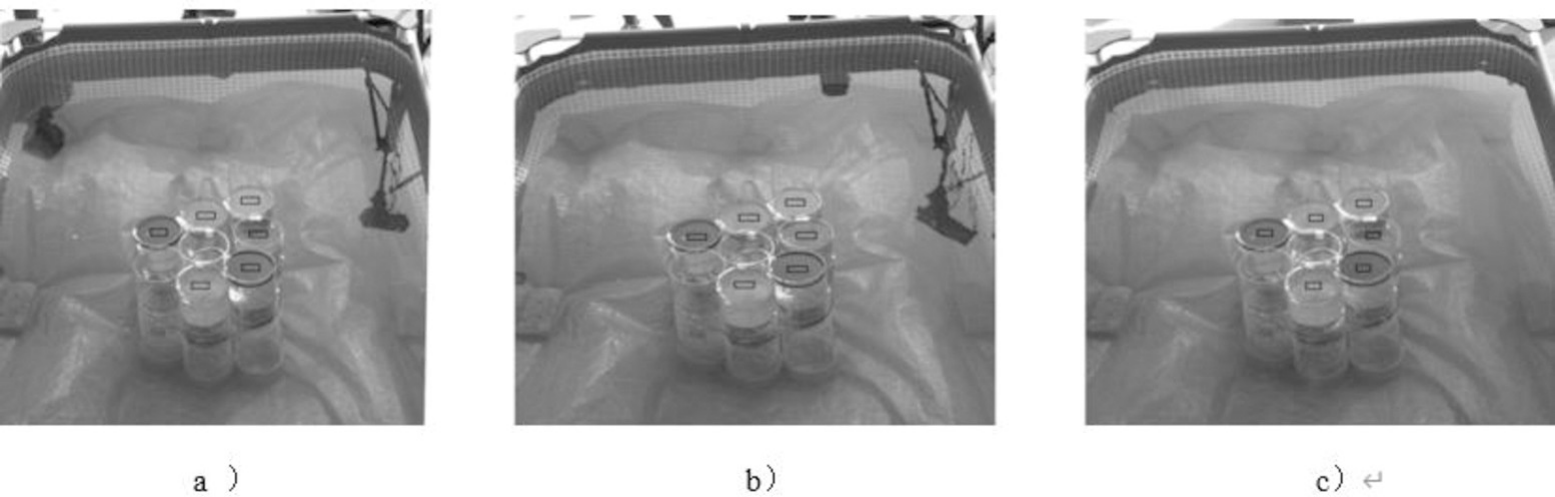
Visible light polarization data at different times (the rectangle in the figure is the part where the gray value is measured). A) 15: 20 b) 15:50 c) 16: 20.

In the non-top-down observation angle, the test conditions were selected as the incidence zenith angle of 51°, the observation zenith angle was 20°, and the relative azimuth angle was 180°, and the experimental oil samples were observed in three groups, and the test oil samples were observed every 30min, as shown in Fig 11. The original image of the obtained oil was processed by using the relevant software, and the polarization characteristic image and polarization degree image of 0°, 45°, 90°, 135° were extracted, and the gray value of the rectangular area with the visible polarization characteristic of each oil product at different times was measured, as shown in Tables 4–6.

**Table 4:** 15:20 grayscale values of visible polarization characteristics of each oil species.

	0°	45°	90°	135°	Polarization degree P	Original S
Heavy Oil	40.1	86.84	156.04	125.86	152.12	101.44
Palm Oil	104.07	138.97	174.78	152.62	64.46	142.14
Crude Oil	40.41	102.34	157.73	112.35	144.97	103.21
Sea Water	76.03	104.76	127.88	107.61	59.64	103.93
Gasoline	88.68	159.83	217.03	164.93	103.48	157.62
Diesel oil	92.92	146.81	198.4	161.51	90.19	149.91

**Table 5:** 15:50 grayscale values of visible polarization characteristics of each oil species.

	0°	45°	90°	135°	Polarization degree P	Original S
Heavy Oil	41.08	88.04	157.86	127.55	151.19	103.74
Palm Oil	104.21	137.59	172.64	151.81	63.00	141.15
Crude Oil	40.31	100.7	155.68	111.09	144.36	102.03
Sea Water	80.18	125.36	161.29	128.87	83.26	124.25
Gasoline	80.3	154.72	216.8	162.89	113.2	154.39
Diesel oil	87.65	142.02	197.44	160.43	96.26	147.44

**Table 6:** 16:20 grayscale values of visible polarization characteristics of each oil species.

	0°	45°	90°	135°	Polarization degree P	Original S
Heavy Oil	40.22	86.06	150.87	120.27	148.13	99.39
Palm Oil	105.2	135.14	162.15	143.82	53.35	136.57
Crude Oil	35.32	87.33	131.46	92.44	141.19	86.27
Sea Water	75.49	90.34	100.21	90.79	32.67	88.46
Gasoline	84.17	144.96	190.88	147.1	95.71	141.8
Diesel oil	93.63	142.63	185.31	151.86	81.55	143.36

**Fig 12:**
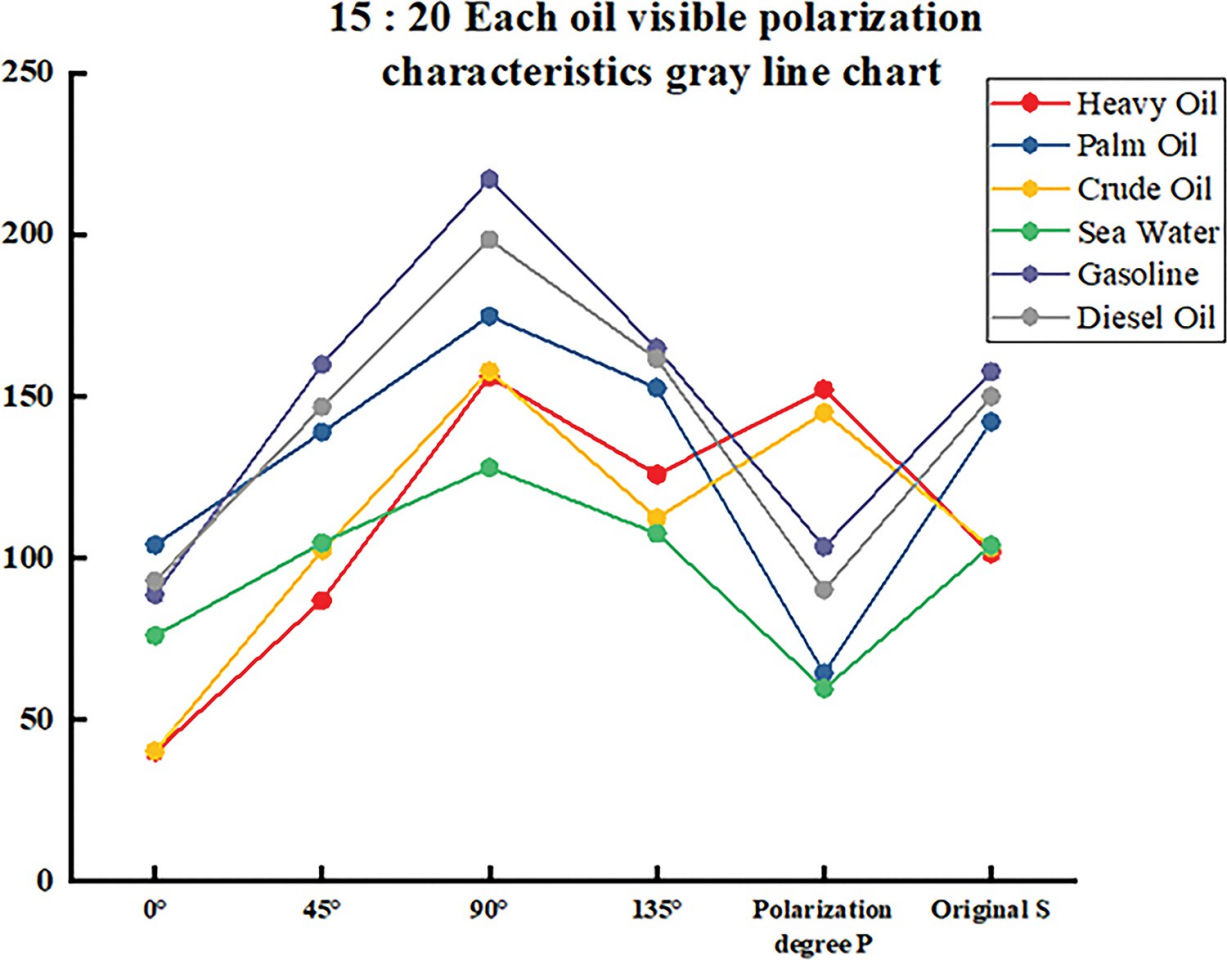
15:20 each oil visible polarization characteristics gray line chart.

**Fig 13:**
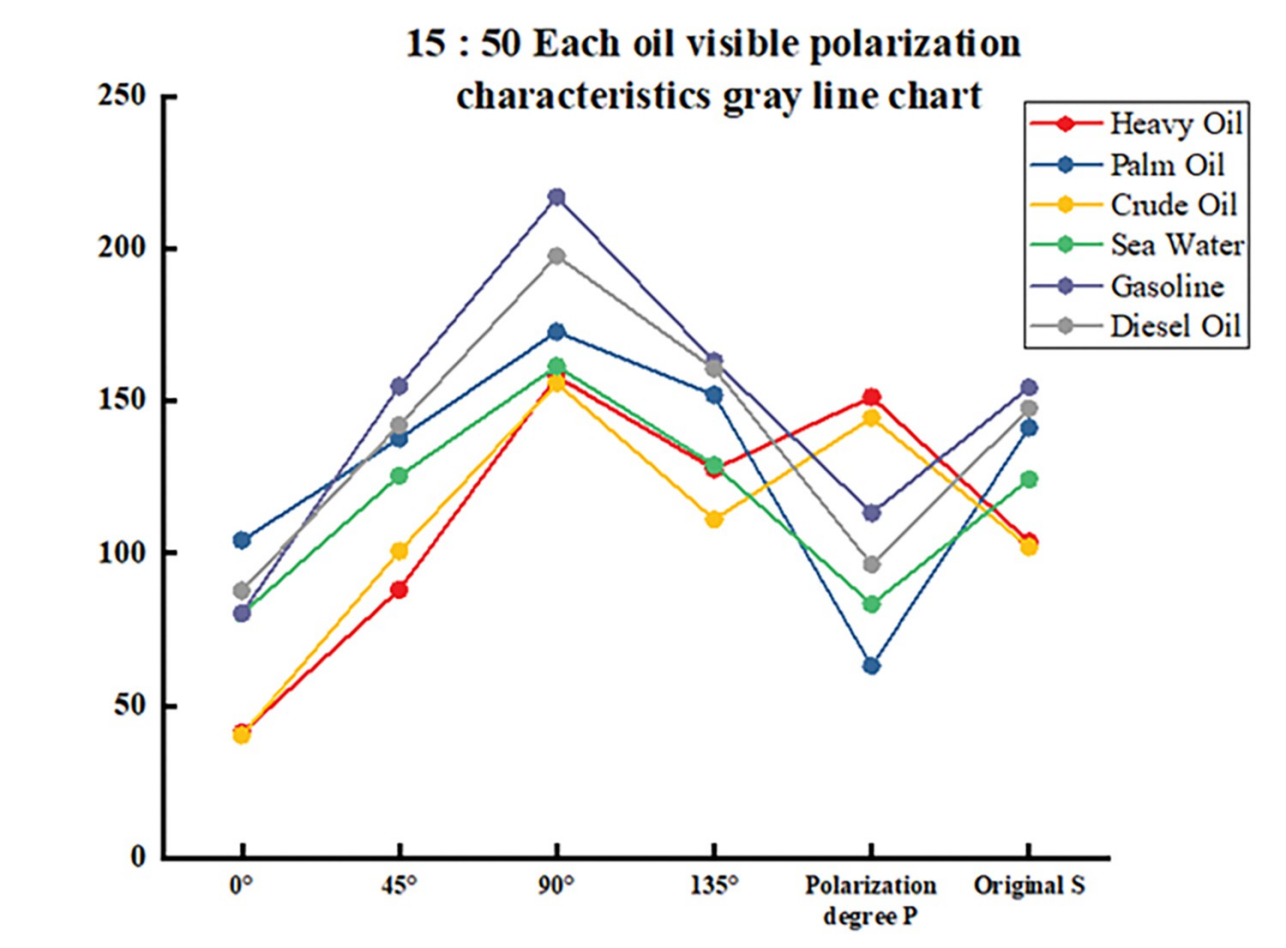
15:50 each oil visible polarization characteristics gray line chart.

**Fig 14:**
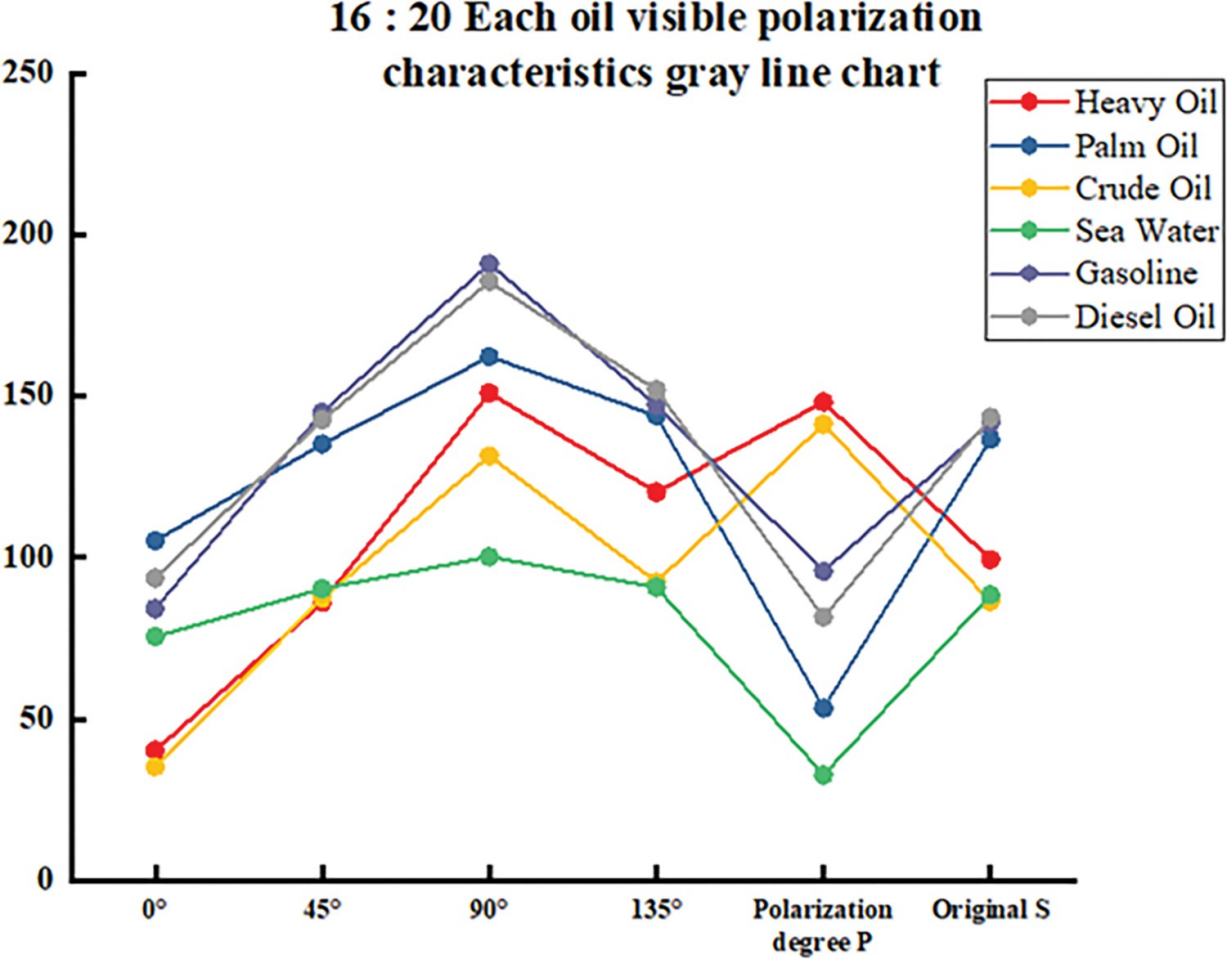
16:20 each oil visible polarization characteristics gray line chart.

The data in the table is plotted as a line graph, as shown in Figs 12–14. It can be seen that the gray value curve of each oil species is tortuous, and the gray value of 90° polarization of the same oil species basically reaches its peak, which is higher than that of other angles and the original image, and is easy to be used as the criterion for oil species differentiation. Although the gray value of the polarization degree image did not reach the highest value, the gray value difference between each oil type and seawater was also obvious. Among them, the effect of palm oil, gasoline and diesel oil was the best, and the effect of heavy oil crude oil was poor. It can be seen that even in the case of fixed observation angle and zenith angle, it can still be used to distinguish oil types.

#### Visible light pBRDF polarization detection experiment

**Fig 15:**
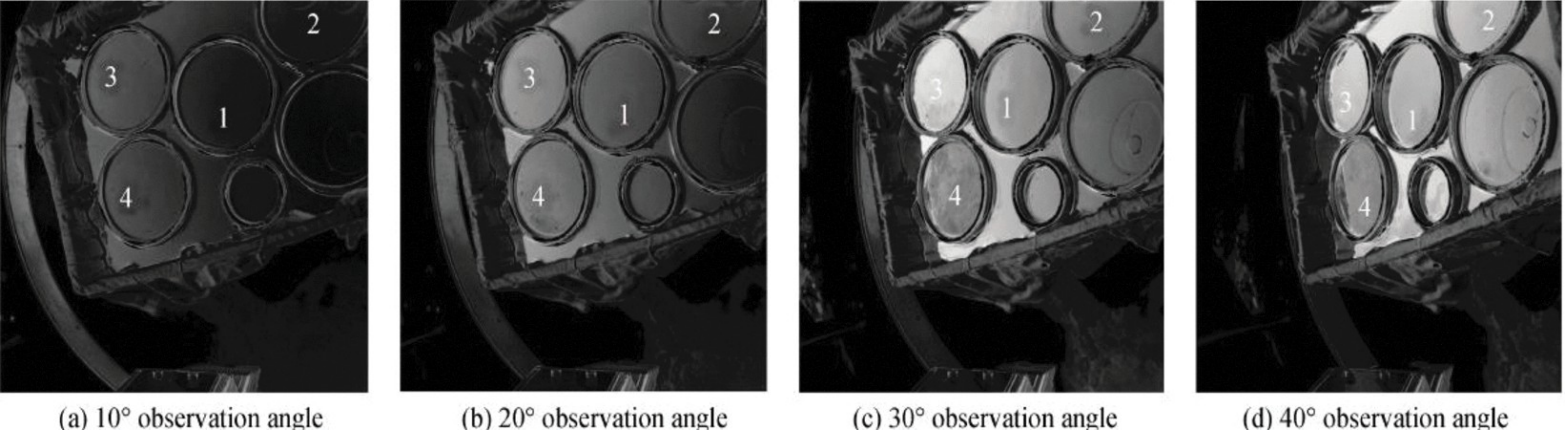
Different observed angle polarization images. a) 10° viewing angle b) 20° viewing angle c) 30° viewing angle d) 40° viewing angle.

**Fig 16:**
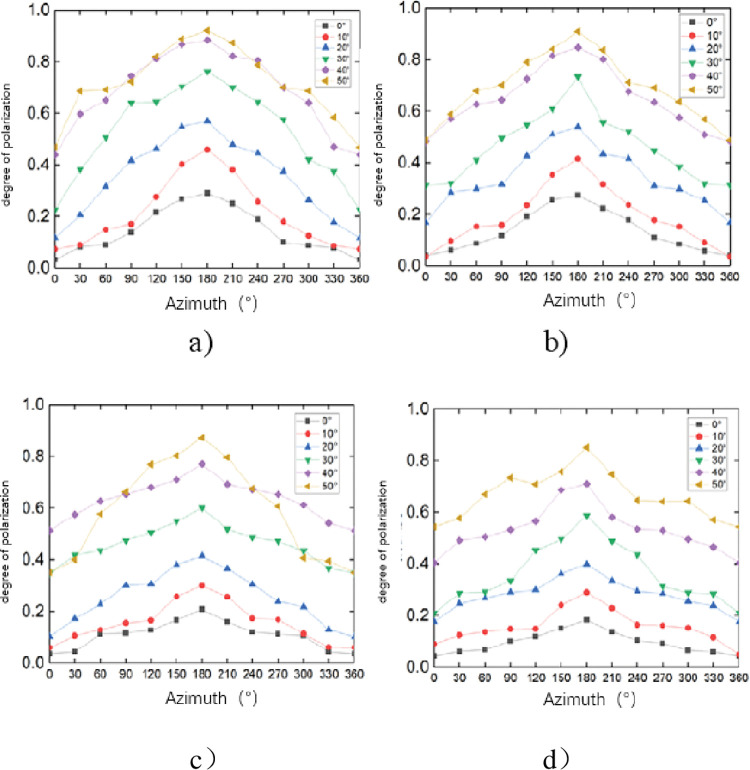
Degrees of polarization of different observation angles of oil spill. a) Fuel b) Crude oil c) Gasoline d) Diesel.

The obtained visible light polarization images of each sample were sorted out, and it was found that the polarization angle image had little effect on distinguishing oil spills, so it was removed and not analyzed. Only the polarization data of the visible polarization image of the sample is analyzed. Some experimental images are shown in Fig 15. Due to the influence of clouds and stray light in the surrounding environment, five sets of polarization data of the same sample will also have different degrees of difference. Therefore, the average value of the five sets of data is taken as the final data for analysis. The visible light polarization of each sample at different azimuth angles is shown in Fig 16.

It can be seen that when the azimuth angle is constant, the visible light polarization of each sample increases with the increase of the observation angle. When the observation angle is constant, the visible light polarization degree of each sample increases first and then decreases with the change of azimuth angle. The polarization degree of 180° azimuth is the highest in the direction of mirror reflection, and the polarization degree of 0° azimuth is the lowest in the direction of solar incidence. During the experiment, factors such as clouds, shadows, flares, and surrounding environments will cause some data to be high or low. It is mainly manifested in the fact that even when the incident angle is close to the Brewster angle, the 180° azimuthal degree of polarization is difficult to approach 1, and there is a big error between the theory and the environmental impact outside the 90° and 270° azimuths.

**Fig 17:**
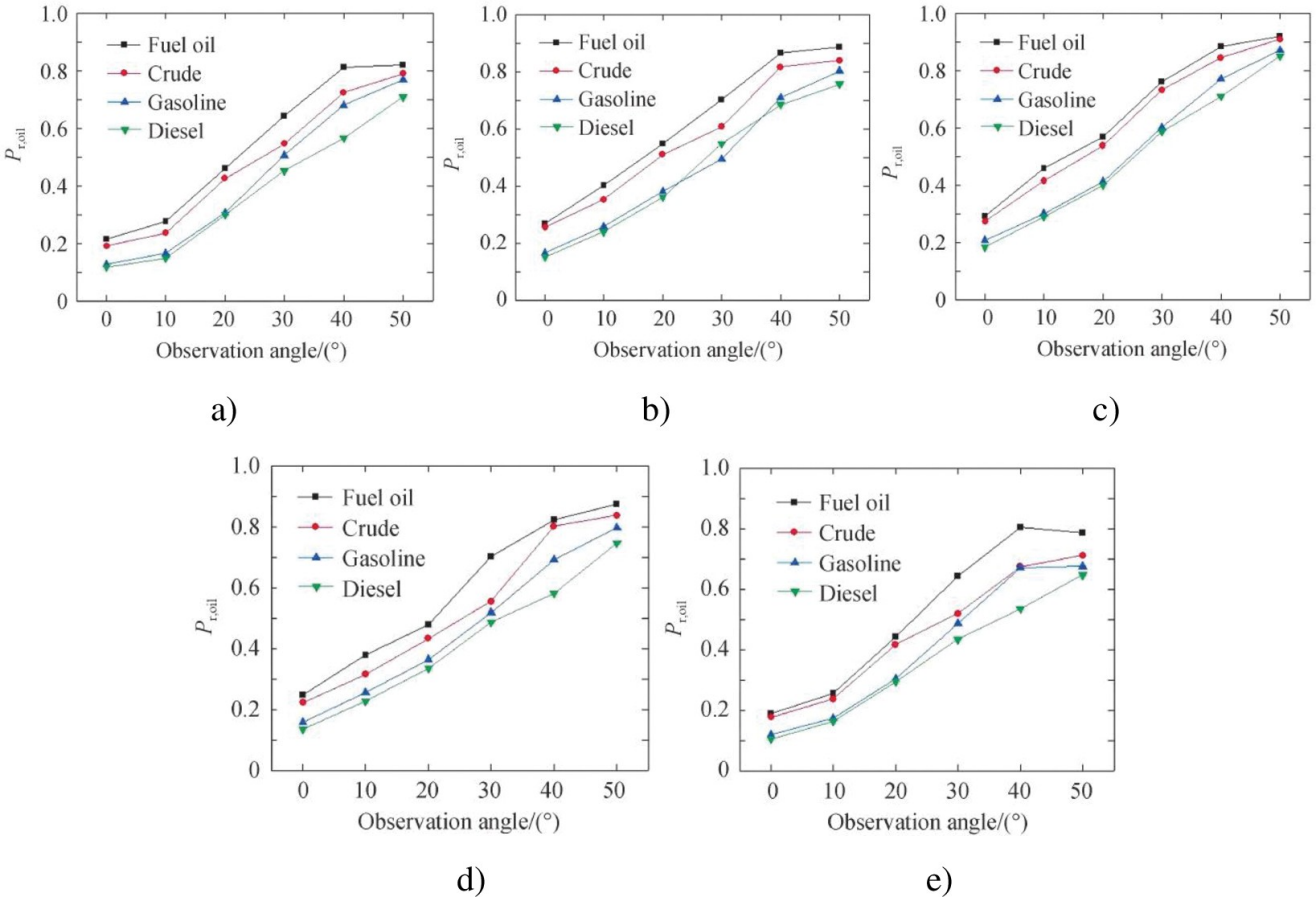
Degrees of visible light polarization of four kinds of oil spill at different azimuth angles. a) 120° orientation b) 150° orientation c) 180° orientation d) 210° orientation e) 240° orientation.

The difference between the samples is analyzed below. From Fig 16, it can be seen that the error is smaller than the theoretical value when the azimuth angle is 120° ~ 240°, indicating that the azimuth angle is the best in this range. Therefore, the visible light polarization curves of four kinds of oil spills at 120°, 150°, 180°, 210° and 240° azimuth angles are given as shown in Fig 17. It can be seen that the degree of polarization of all samples increases with the increase of observation angle when the azimuth angle is 120° ~ 240°. The visible light polarization degree from high to low is fuel oil, crude oil, gasoline and diesel. The difference between heavy oil and light oil is large, the difference between fuel oil and crude oil is small, and the difference between gasoline and diesel is small. The distinction of all samples can be basically realized.

**Fig 18:**
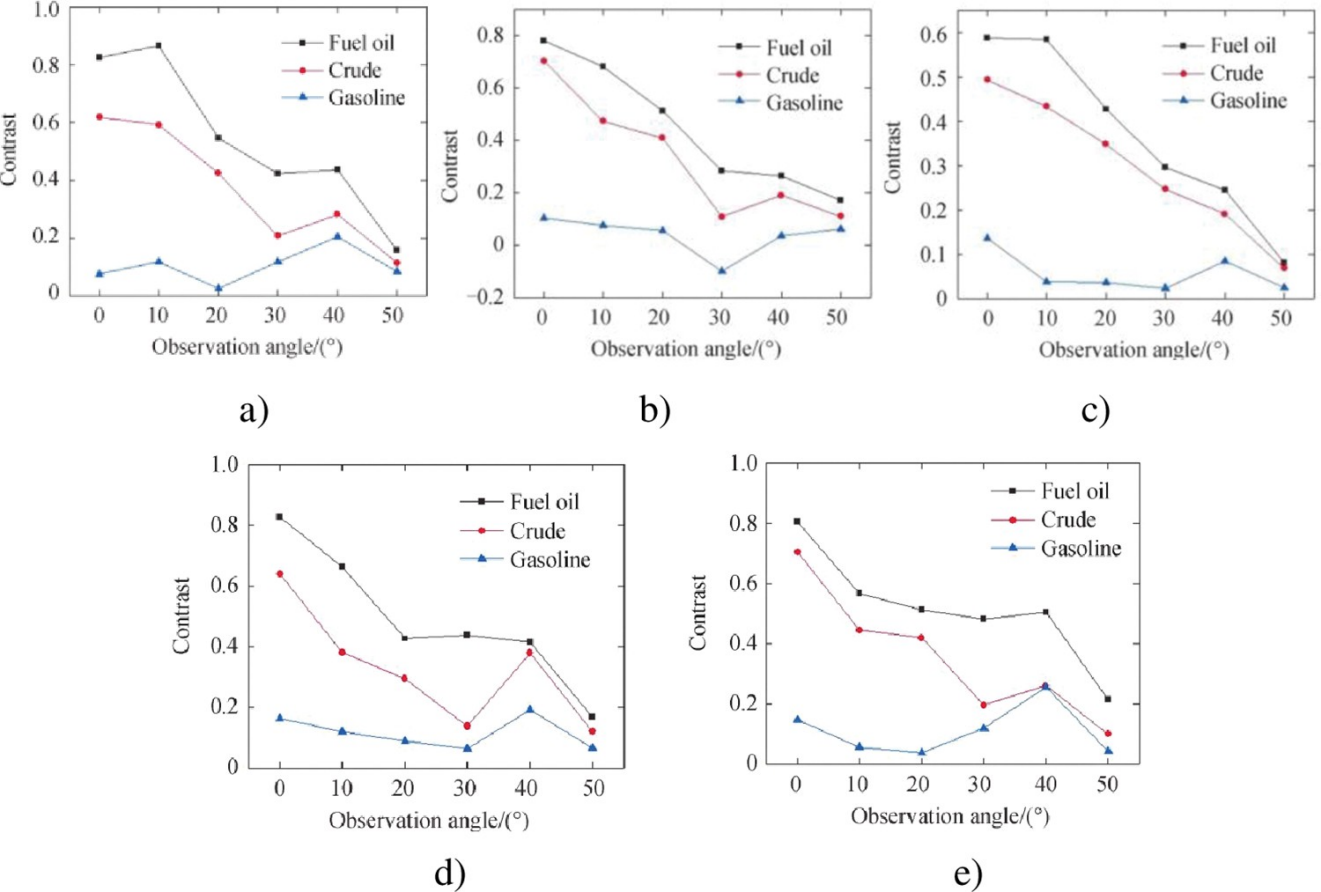
Visible polarization contrast of fuel, crude oil and gasoline relative to diesel at different azimuth angles. a) 120° orientation b) 150° orientation c) 180° orientation d) 210° orientation e) 240° orientation.

In order to show the degree of differentiation of each oil, the polarization contrast of each oil relative to diesel is calculated with diesel as reference. Fig 18 shows the visible polarization contrast of fuel, crude oil, and gasoline relative to diesel at an azimuth angle of 120° to 240°. Obviously, when the observation angle increases, the contrast generally shows a decreasing trend. It shows that when the observation angle is close to the Brewster angle, the degree of polarization of each oil is very high, but it is very close, resulting in a decrease in contrast and a relatively poor distinction. The contrast range of fuel relative to diesel is about [0.2,0.8], the contrast range of crude oil relative to diesel is about [0.15,0.7], and the contrast range of gasoline relative to diesel is about [0.05,0.15]. Overall contrast is generally higher than 0.05, distinguish effect is good.

## Conclusions

The influence of different azimuth angles and zenith angles on the polarization degree of oil spill was analyzed by using the visible light polarization detection test of oil spill. Theoretical simulations and experiments show that the influence of azimuth angle and zenith angle on the polarization degree is greater than that of refractive index. The polarization degree changes of samples with different azimuth angles and zenith angles were analyzed by using the oil spill visible light polarization detection experiment. When the azimuth angle is 120° ~ 240°, the polarization degree of visible light observed at the same angle from high to low is fuel oil, crude oil, gasoline and diesel, and the polarization degree contrast between each oil type is generally higher than 0.05. It shows that it is feasible to distinguish oil species by polarization. The marine environment is complex and changeable, and there are many factors affecting the results. Considering the external environmental factors, emulsification of seawater oil, outdoor humidity, wind speed and other reasons, the outdoor measurement error will occur. It is difficult to find the change rule of each sample only from the test. In the future, it is necessary to further explore the influence of spectral band, temperature and environmental changes on the polarization characteristics of samples, establish a more perfect theoretical model, and improve the ability to identify oil spill types.

## Supporting information

S1 File Experimental data of oil leakage DOP at different viewing angles. (DOCX)

S2 File Experimental data of DOP for oil leakage in four different azimuthal angles. (DOCX)

S3 File Experimental data of visible polarization contrast. (DOCX)
