# Supplementary material for: Detection and identification of oil spill species based on polarization information
Source: PLoS One. 2023 Nov 30;18(11):e0291553. doi: 10.1371/journal.pone.0291553 (PMC10688671; doi:10.1371/journal.pone.0291553)

The following data is available in the article:

Experimental data in Fig16 (a) ~ (d) in this paper（0°-50° is observation angle, 0°-360° is azimuth）


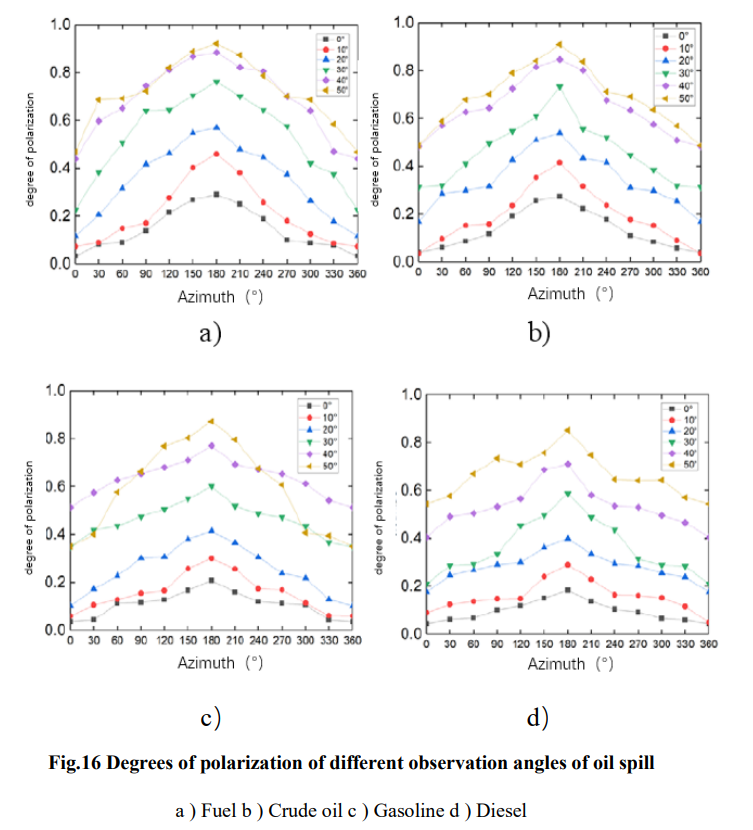


Table 1. Polarization degree of oil spill at different observation angles of Fuel

|  | 0° | 30° | 60° | 90° | 120° | 150° | 180° | 210° | 240° | 270° | 300° | 330° | 360° |
| --- | --- | --- | --- | --- | --- | --- | --- | --- | --- | --- | --- | --- | --- |
| 0° | 0.029 | 0.071 | 0.084 | 0.135 | 0.215 | 0.269 | 0.283 | 0.244 | 0.183 | 0.096 | 0.082 | 0.067 | 0.029 |
| 10° | 0.066 | 0.087 | 0.082 | 0.167 | 0.274 | 0.400 | 0.452 | 0.375 | 0.254 | 0.176 | 0.121 | 0.080 | 0.066 |
| 20° | 0.110 | 0.201 | 0.309 | 0.409 | 0.459 | 0.542 | 0.569 | 0.475 | 0.439 | 0.370 | 0.260 | 0.171 | 0.107 |
| 30° | 0.224 | 0.375 | 0.503 | 0.637 | 0.644 | 0.702 | 0.763 | 0.699 | 0.644 | 0.573 | 0.413 | 0.373 | 0.224 |
| 40° | 0.435 | 0.591 | 0.650 | 0.742 | 0.803 | 0.862 | 0.880 | 0.821 | 0.808 | 0.696 | 0.635 | 0.466 | 0.432 |
| 50° | 0.468 | 0.683 | 0.685 | 0.721 | 0.794 | 0.885 | 0.918 | 0.870 | 0.785 | 0.696 | 0.683 | 0.583 | 0.466 |

Table 2. Polarization degree of oil spill at different observation angles of Crude oil

|  | 0° | 30° | 60° | 90° | 120° | 150° | 180° | 210° | 240° | 270° | 300° | 330° | 360° |
| --- | --- | --- | --- | --- | --- | --- | --- | --- | --- | --- | --- | --- | --- |
| 0° | 0.030 | 0.060 | 0.087 | 0..115 | 0.188 | 0.254 | 0.273 | 0.218 | 0.176 | 0.106 | 0.085 | 0.060 | 0.045 |
| 10° | 0.028 | 0.096 | 0.147 | 0.152 | 0.235 | 0.350 | 0.408 | 0.315 | 0.235 | 0.177 | 0.147 | 0.089 | 0.033 |
| 20° | 0.162 | 0.281 | 0.293 | 0.315 | 0.427 | 0.507 | 0.534 | 0.430 | 0.410 | 0.310 | 0.291 | 0.249 | 0.162 |
| 30° | 0.315 | 0.318 | 0.408 | 0.490 | 0.549 | 0.607 | 0.734 | 0.554 | 0.515 | 0.439 | 0.381 | 0.320 | 0.310 |
| 40° | 0.478 | 0.563 | 0.622 | 0.641 | 0.724 | 0.811 | 0.848 | 0.797 | 0.673 | 0.629 | 0.568 | 0.507 | 0.480 |
| 50° | 0.488 | 0.588 | 0.677 | 0.702 | 0.787 | 0.838 | 0.906 | 0.836 | 0.709 | 0.690 | 0.631 | 0.568 | 0.480 |

Table 3. Polarization degree of oil spill at different observation angles of Gasoline

|  | 0° | 30° | 60° | 90° | 120° | 150° | 180° | 210° | 240° | 270° | 300° | 330° | 360° |
| --- | --- | --- | --- | --- | --- | --- | --- | --- | --- | --- | --- | --- | --- |
| 0° | 0.037 | 0.046 | 0.109 | 0.115 | 0.129 | 0.172 | 0.208 | 0.158 | 0.122 | 0.115 | 0.102 | 0.045 | 0.037 |
| 10° | 0.056 | 0.106 | 0.129 | 0.158 | 0.172 | 0.257 | 0.300 | 0.257 | 0.175 | 0.168 | 0.112 | 0.060 | 0.060 |
| 20° | 0.102 | 0.172 | 0.227 | 0.303 | 0.306 | 0.379 | 0.415 | 0.365 | 0.306 | 0.241 | 0.221 | 0.129 | 0.102 |
| 30° | 0.349 | 0.415 | 0.438 | 0.474 | 0.507 | 0.550 | 0.599 | 0.520 | 0.487 | 0.474 | 0.434 | 0.362 | 0.349 |
| 40° | 0.513 | 0.572 | 0.622 | 0.658 | 0.681 | 0.711 | 0.767 | 0.691 | 0.671 | 0.648 | 0.612 | 0.543 | 0.507 |
| 50° | 0.349 | 0.395 | 0.572 | 0.658 | 0.767 | 0.799 | 0.869 | 0.796 | 0.671 | 0.603 | 0.408 | 0.392 | 0.349 |

Table 4. Polarization degree of oil spill at different observation angles of Diesel

|  | 0° | 30° | 60° | 90° | 120° | 150° | 180° | 210° | 240° | 270° | 300° | 330° | 360° |
| --- | --- | --- | --- | --- | --- | --- | --- | --- | --- | --- | --- | --- | --- |
| 0° | 0.045 | 0.058 | 0.064 | 0.099 | 0.117 | 0.148 | 0.186 | 0.133 | 0.101 | 0.089 | 0.061 | 0.054 | 0.036 |
| 10° | 0.089 | 0.123 | 0.136 | 0.148 | 0.149 | 0.242 | 0.286 | 0.223 | 0.161 | 0.161 | 0.148 | 0.114 | 0.036 |
| 20° | 0.170 | 0.245 | 0.264 | 0.289 | 0.299 | 0.358 | 0.399 | 0.330 | 0.289 | 0.283 | 0.252 | 0.236 | 0.174 |
| 30° | 0.211 | 0.283 | 0.292 | 0.333 | 0.449 | 0.496 | 0.580 | 0.489 | 0.433 | 0.308 | 0.289 | 0.280 | 0.205 |
| 40° | 0.399 | 0.487 | 0.502 | 0.533 | 0.565 | 0.684 | 0.705 | 0.574 | 0.536 | 0.521 | 0.493 | 0.458 | 0.396 |
| 50° | 0.540 | 0.574 | 0.665 | 0.731 | 0.702 | 0.756 | 0.849 | 0.743 | 0.646 | 0.637 | 0.640 | 0.568 | 0.536 |


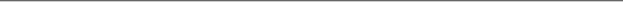

Supplement: S1 File — (DOCX) [file pone.0291553.s001.docx]
