# Supplementary material for: Detection and identification of oil spill species based on polarization information
Source: PLoS One. 2023 Nov 30;18(11):e0291553. doi: 10.1371/journal.pone.0291553 (PMC10688671; doi:10.1371/journal.pone.0291553)
Supplement: S2 File — (DOCX) [file pone.0291553.s002.docx]

Experimental data in Fig18 (a) ~ (e) in this paper:


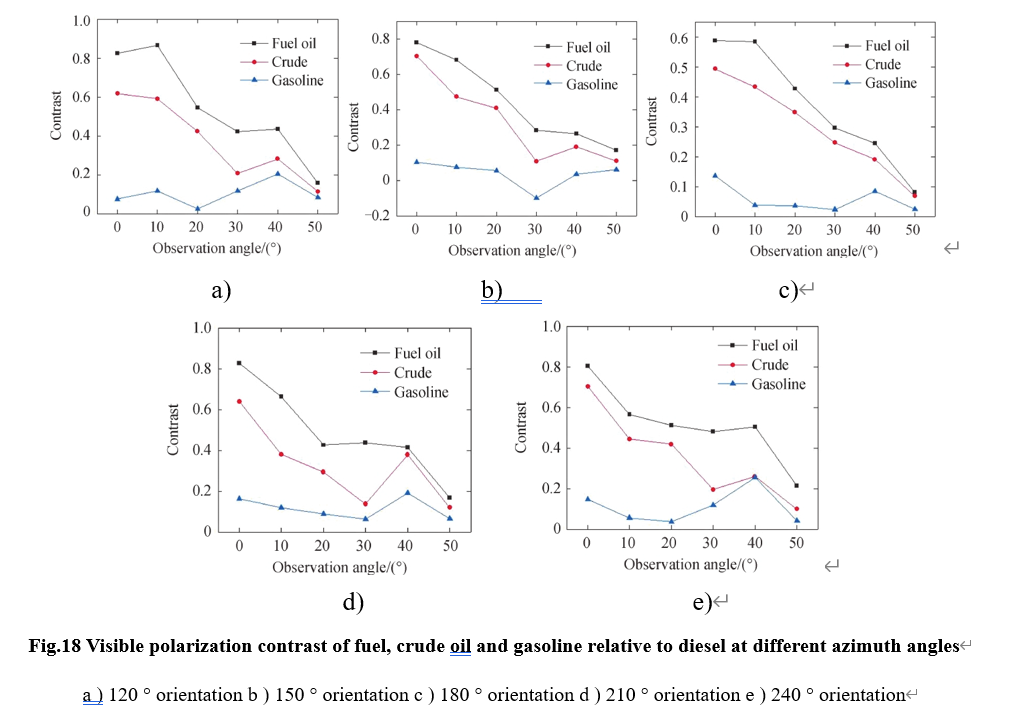


Table 10 Visible polarization comparison of fuel oil, crude oil and gasoline with respect to diesel at 120° azimuth

| kind of oil | 0° | 10° | 20° | 30° | 40° | 50° |
| --- | --- | --- | --- | --- | --- | --- |
| Fuel oil | 0.824 | 0.866 | 0.542 | 0.419 | 0.439 | 0.160 |
| Crude | 0.618 | 0.595 | 0.422 | 0.207 | 0.288 | 0.112 |
| Gasoline | 0.070 | 0.112 | 0.023 | 0.118 | 0.204 | 0.079 |

Table 11 Visible polarization comparison of fuel oil, crude oil and gasoline with respect to diesel at 150° azimuth

observation

angle

observation

angle

| kind of oil | 0° | 10° | 20° | 30° | 40° | 50° |
| --- | --- | --- | --- | --- | --- | --- |
| Fuel oil | 0.779 | 0.683 | 0.516 | 0.282 | 0.265 | 0.172 |
| Crude | 0.703 | 0.479 | 0.406 | 0.109 | 0.189 | 0.109 |
| Gasoline | 0.102 | 0.076 | 0.059 | -0.100 | 0.032 | 0.061 |

Table 12 Visible polarization comparison of fuel oil, crude oil and gasoline with respect to diesel at 180° azimuth

observation

angle

| kind of oil | 0° | 10° | 20° | 30° | 40° | 50° |
| --- | --- | --- | --- | --- | --- | --- |
| Fuel oil | 0.586 | 0.581 | 0.427 | 0.298 | 0.246 | 0.084 |
| Crude | 0.491 | 0.434 | 0.350 | 0.248 | 0.191 | 0.070 |
| Gasoline | 0.138 | 0.041 | 0.036 | 0.027 | 0.087 | 0.023 |

Table 13 Visible polarization comparison of fuel oil, crude oil and gasoline with respect to diesel at 210° azimuth

| kind of oil | 0° | 10° | 20° | 30° | 40° | 50° |
| --- | --- | --- | --- | --- | --- | --- |
| Fuel oil | 0.833 | 0.666 | 0.428 | 0.439 | 0.417 | 0.168 |
| Crude | 0.639 | 0.382 | 0.297 | 0.138 | 0.382 | 0.119 |
| Gasoline | 0.160 | 0.119 | 0.089 | 0.062 | 0.192 | 0.067 |

Table 14 Visible polarization comparison of fuel oil, crude oil and gasoline with respect to diesel at 240° azimuth

observation

angle

observation

angle

| kind of oil | 0° | 10° | 20° | 30° | 40° | 50° |
| --- | --- | --- | --- | --- | --- | --- |
| Fuel oil | 0.807 | 0.570 | 0.518 | 0.483 | 0.502 | 0.216 |
| Crude | 0.705 | 0.443 | 0.419 | 0.197 | 0.257 | 0.098 |
| Gasoline | 0.146 | 0.057 | 0.041 | 0.119 | 0.257 | 0.041 |
