# Supplementary material for: Detection and identification of oil spill species based on polarization information
Source: PLoS One. 2023 Nov 30;18(11):e0291553. doi: 10.1371/journal.pone.0291553 (PMC10688671; doi:10.1371/journal.pone.0291553)

Experimental data in Fig17 (a) ~ (e) in this paper:


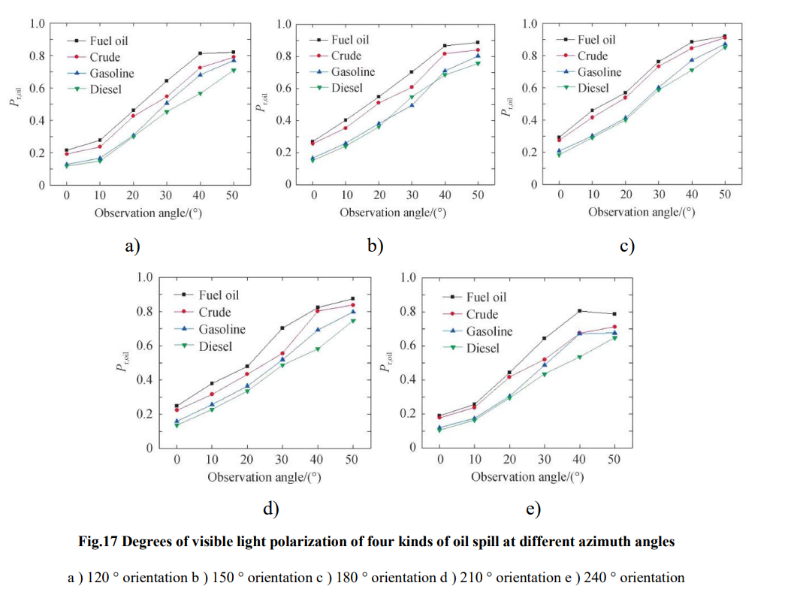


Table 5. Visible light polarization of four oil spills at 120° azimuth

observation

angle

| kind of oil | 0° | 10° | 20° | 30° | 40° | 50° |
| --- | --- | --- | --- | --- | --- | --- |
| Fuel oil | 0.217 | 0.277 | 0.459 | 0.643 | 0.812 | 0.818 |
| Crude | 0.190 | 0.232 | 0.425 | 0.543 | 0.727 | 0.794 |
| Gasoline | 0.113 | 0.163 | 0.302 | 0.501 | 0.676 | 0.764 |
| Diesel | 0.114 | 0.147 | 0.298 | 0.452 | 0.567 | 0.709 |

Table 6. Visible light polarization of four oil spills at 150° azimuth

observation

angle

| kind of oil | 0° | 10° | 20° | 30° | 40° | 50° |
| --- | --- | --- | --- | --- | --- | --- |
| Fuel oil | 0.263 | 0.401 | 0.545 | 0.697 | 0.867 | 0.883 |
| Crude | 0.262 | 0.356 | 0.511 | 0.604 | 0.811 | 0.843 |
| Gasoline | 0.164 | 0.257 | 0.380 | 0.489 | 0.705 | 0.793 |
| Diesel | 0.164 | 0.246 | 0.361 | 0.540 | 0.686 | 0.753 |

Table 7. Visible light polarization of four oil spills at 180° azimuth

| kind of oil | 0° | 10° | 20° | 30° | 40° | 50° |
| --- | --- | --- | --- | --- | --- | --- |
| Fuel oil | 0.289 | 0.457 | 0.565 | 0.758 | 0.882 | 0.921 |
| Crude | 0.273 | 0.413 | 0.534 | 0.728 | 0.842 | 0.903 |
| Gasoline | 0.201 | 0.297 | 0.406 | 0.595 | 0.767 | 0.870 |
| Diesel | 0.185 | 0.296 | 0.397 | 0.586 | 0.709 | 0.847 |

Table 8. Visible light polarization of four oil spills at 210° azimuth

observation

angle

observation

angle

| kind of oil | 0° | 10° | 20° | 30° | 40° | 50° |
| --- | --- | --- | --- | --- | --- | --- |
| Fuel oil | 0.247 | 0.378 | 0.483 | 0.703 | 0.828 | 0.875 |
| Crude | 0.219 | 0.317 | 0.433 | 0.553 | 0.805 | 0.836 |
| Gasoline | 0.158 | 0.253 | 0.364 | 0.516 | 0.697 | 0.800 |
| Diesel | 0.136 | 0.228 | 0.336 | 0.478 | 0.580 | 0.744 |

Table 9. Visible light polarization of four oil spills at 240° azimuth

observation

angle

| kind of oil | 0° | 10° | 20° | 30° | 40° | 50° |
| --- | --- | --- | --- | --- | --- | --- |
| Fuel oil | 0.189 | 0.254 | 0.445 | 0.642 | 0.803 | 0.787 |
| Crude | 0.177 | 0.236 | 0.417 | 0.524 | 0.676 | 0.714 |
| Gasoline | 0.121 | 0.171 | 0.304 | 0.488 | 0.676 | 0.678 |
| Diesel | 0.110 | 0.162 | 0.287 | 0.429 | 0.535 | 0.646 |


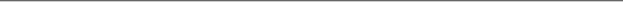

Supplement: S3 File — (DOCX) [file pone.0291553.s003.docx]
